# Increase in IFNγ^−^IL-2^+^ Cells in Recent Human CD4 T Cell Responses to 2009 Pandemic H1N1 Influenza

**DOI:** 10.1371/journal.pone.0057275

**Published:** 2013-03-20

**Authors:** Jason M. Weaver, Hongmei Yang, David Roumanes, F. Eun-Hyung Lee, Hulin Wu, John J. Treanor, Tim R. Mosmann

**Affiliations:** 1 David H. Smith Center for Vaccine Biology and Immunology, University of Rochester Medical Center, Rochester, New York, United States of America; 2 Department of Biostatistics and Computational Biology, University of Rochester Medical Center, Rochester, New York, United States of America; 3 Division of Pulmonary Medicine, Department of Medicine, University of Rochester Medical Center, Rochester, New York, United States of America; 4 Division of Infectious Diseases, Department of Medicine, University of Rochester Medical Center, Rochester, New York, United States of America; St. Jude Children's Research Hospital, United States of America

## Abstract

Human CD4 T cell recall responses to influenza virus are strongly biased towards Type 1 cytokines, producing IFNγ, IL-2 and TNFα. We have now examined the effector phenotypes of CD4 T cells in more detail, particularly focusing on differences between recent versus long-term, multiply-boosted responses. Peptides spanning the proteome of temporally distinct influenza viruses were distributed into pools enriched for cross-reactivity to different influenza strains, and used to stimulate antigen-specific CD4 T cells representing recent or long-term memory. In the general population, peptides unique to the long-circulating influenza A/New Caledonia/20/99 (H1N1) induced Th1-like responses biased toward the expression of IFNγ^+^TNFα^+^ CD4 T cells. In contrast, peptide pools enriched for non-cross-reactive peptides of the pandemic influenza A/California/04/09 (H1N1) induced more IFNγ^−^IL-2^+^TNFα^+^ T cells, similar to the IFNγ^−^IL-2^+^ non-polarized, primed precursor T cells (Thpp) that are a predominant response to protein vaccination. These results were confirmed in a second study that compared samples taken before the 2009 pandemic to samples taken one month after PCR-confirmed A/California/04/09 infection. There were striking increases in influenza-specific TNFα^+^, IFNγ^+^, and IL-2^+^ cells in the post-infection samples. Importantly, peptides enriched for non-cross-reactive A/California/04/09 specificities induced a higher proportion of Thpp-like IFNγ^−^IL-2^+^TNFα^+^ CD4 T cells than peptide pools cross-reactive with previous influenza strains, which induced more Th1 (IFNγ^+^TNFα^+^) responses. These IFNγ^−^IL-2^+^TNFα^+^ CD4 T cells may be an important target population for vaccination regimens, as these cells are induced upon infection, may have high proliferative potential, and may play a role in providing future effector cells during subsequent infections.

## Introduction

Although antibodies are undoubtedly important for protection against influenza virus infection, there is increasing interest in the potential value of CD8 and CD4 T cell responses [1]. Potential T cell mechanisms include help for antibody protection, as well as inflammation and direct cytotoxicity mediated by both CD4 and CD8 T cells. As T cell responses may blunt the progress of influenza infection rather than prevent the initial infection outright, T cell protection may be more useful for reducing severity. Live attenuated influenza vaccine may induce more T cell but less antibody immunity than TIV [2], yet LAIV is still an effective vaccine, and may be more effective in a year when the vaccine and circulating strains are less well-matched, consistent with broader cross-reactivity of T cells than antibody [3]. A recent study suggested that CD4 T cell responses correlated with protection in a challenge model [4], so measuring T cell responses is important for evaluating future vaccine candidates.

The human CD4 T cell memory response to influenza is normally skewed strongly to the Th1 pattern of cytokine expression, including mainly cells secreting IFNγ, TNFα and IL-2 but not IL-4 [5]–[8]. This pattern is also induced by additional viruses and intracellular bacteria, but contrasts with the Th2 (IL-4, IL-5) effector cytokine response patterns of T cells specific for helminths, and the Th17 (IL-17) responses induced by some bacterial and fungal pathogens (reviewed in Zielinski et al. [9]). We also identified an uncommitted subset of antigen-specific memory T cells in both mice [10]–[13] and humans [5]. These T helper primed, precursor (Thpp) cells do not express effector cytokines such as IL-4, IFNγ or IL-17, but individual cells are uncommitted, and can differentiate into either Th1 or Th2 T cells in response to the appropriate signals *in vitro*. Thpp cells account for a substantial proportion of the memory response induced by protein vaccines for tetanus, diphtheria and Hepatitis B [5], and overlap at least partially with the central memory (Tcm) phenotype of lymphoid tissue-homing cells [14]–[16]. Although some definitions of Tcm cells include IFNγ-producing T cells [17]–[19], these cells are not included within the Thpp population.

Within the overall Type 1 cytokine pattern, ‘multifunctional’ cells simultaneously produce a higher number of the Th1 cytokines, and in some cases are associated with more effective immune responses in human HIV infection [20], human vaccinations [21]–[23] and mouse vaccination for either *L. major* [24] or *M. tuberculosis* [25]. Although multifunctional effector T cells may have advantages in the short term for pathogen eradication, Thpp cells have a greater proliferative potential [26] and may be valuable for preserving long-term memory by serving as the major reservoir of future effector cells against subsequent infections [27]. Thus the balance between different subsets of T cells may be important to preserve the capabilities for both immediate protection as well as long-term maintenance of memory.

Humans normally encounter influenza antigens many times by infection and/or vaccination, and T cell responses to influenza are normally detectable in all adults. However, there is little understanding on how distinct subsets of memory cells are generated and maintained over long periods of time in humans. The pandemic H1N1 influenza in 2009 (A/California/04/09, CA/09) is a novel quadruple reassortant that provided the opportunity to assess the functional quality of newly formed memory CD4 T cells in the adult human population. The CA/09 strain contains substantial numbers of epitopes not shared with recent influenza strains since the 1976 vaccine [28], so most young adults had little reactivity against these epitopes before exposure to CA/09. We therefore analyzed the CD4 T cell responses unique to recent and long-circulating strains of influenza to determine the impact of time on the quality (i.e. cytokine patterns) of memory T cells.

Overlapping peptides from chronologically distinct influenza viruses were pooled into groups designed to enrich for responses against a recent antigen encounter (CA/09) or a long-circulating influenza strain (A/New Caledonia/20/99, NC/99). CD4 T cell responses to recent exposure included relatively more IFNγ^−^IL-2^+^TNFα^+^ producing cells in comparison to long-term T cell responses in the general population. In a second study, this preference also occurred in responses sampled shortly after documented CA/09 infection. These results reveal an unexpected diversity in the quality of the human CD4 T cell response to influenza, and may have implications for vaccine design to optimize effector and long-term memory responses.

## Materials and Methods

### Subjects

#### Study 1

Twenty-seven healthy subjects, ages 19–49 years (median 33±4.5 years), had blood samples collected between 07/2009–10/2010. No subjects reported influenza-like illnesses (ILI), and 5 reported immunization with the H1N1 vaccine. Fourteen subjects (including all of the vaccinated subjects) had a detectable antibody titer (≥1∶20) to the hemagglutinin of A/California/04/09 at the time of blood draw.

#### Study 2

Blood samples were collected and frozen from ten healthy subjects, ages 22–62 years (median 43±15.5 years) in the fall of 2008 i.e. before the 2009 influenza pandemic. Eight subjects with PCR confirmed A/California/04/2009 influenza infection, ages 29–52 years (median 44±8.6 years), were enrolled on average on day 5 of illness during the winter of 2009/2010. Prior influenza vaccination history and ILI in the recent past was obtained.

### Ethics Statement

All procedures were approved by the Research Subjects Review Board at the University of Rochester Medical Center, Rochester, New York.

### Sample collection

Peripheral blood mononuclear cells (PBMC) were isolated from sodium heparinized peripheral blood by ficoll-hypaque gradient centrifugation, washed and prepared for freezing by the sample-processing core. PBMC were cryopreserved in 90% FBS and 10% DMSO (Sigma-Aldrich, St. Louis, MO) and frozen to −80°C using an isopropanol-filled, controlled-rate freezing device. After 24–48 hrs at −80°C, the vials were transferred into liquid nitrogen for long-term storage.

### Peptide antigens

Individual peptides consisting of 15–17 amino acid residues, offset by 5 amino acids were designed to span the proteome of A/California/04/2009, hemagglutinin (HA) and neuraminidase (NA) from A/Brisbane/59/2007, and nucleoprotein (NP), polymerase acid protein (PA), polymerase basic proteins (PB1 and PB2), matrix proteins (M1 and M2), non-structural proteins (NS1 and NS2) from A/Puerto Rico/8/1934 (PR8) (synthesized by Mimotopes). The following CD4 T cell-restricted epitopes from Tetanus: L31-50, L271-290, L286-305, H56-75, H116-135, H131-150, H161-180, H176-195, H191-210, H251-270, H373-387, H431-450, H491-510, H566-585, H731-750, H791-810; where L and H are Light and Heavy chains, respectively [29]–[31], were synthesized by Mimotopes. The following reagents were obtained through the NIH Biodefense and Emerging Infections Research Resources Repository, NIAID, NIH: Peptide Array, Influenza Virus A/New Caledonia/20/99 (H1N1) HA Protein, NR-2602, NA protein, NR-2606, Influenza Virus A/New York/348/03 (H1N1) Nucleocapsid protein, NR-2611, PA Protein, NR-2618, PB1 Protein, NR-2617, PB2 Protein, NR-2616, Matrix Protein 1, NR-2613, Matrix Protein 2, NR-2614, Nonstructural Protein 2, NR-2615, Influenza Virus A/New York/444/01 Nonstructural Protein 1, NR-2612. As the structural proteins of A/New York/348/03, A/New York/444/01 and A/New Caledonia/20/99 are 99.7% identical and 99.9% similar, the A/New Caledonia/20/99 HA/NA, and A/New York/348/03 and A/New York/444/01 internal protein peptides are described in aggregate as the New Caledonia (NC/99) peptides in the experiments described here. Peptides were resuspended in DMSO and 5 mM 2-mercaptoethanol at 20 mg/ml or 50 mg/ml, and assembled into pools of peptides representing levels of cross-reactivity across different virus strains (see Results). Stocks of peptide pools were diluted into dH2O at a final concentration of 0.02 mg/ml/peptide for influenza and 1 mg/ml/peptide for tetanus. Influenza peptide pools were used at a final concentration of 0.1 µg/ml/peptide and the tetanus peptide pool was used at a final concentration of 3 µg/ml/peptide. In preliminary experiments we compared individual proteins (∼19–200 peptides/pool) to the influenza proteome (737 peptides) and showed that in general smaller pools give slightly greater numbers of responding T cells. In dose response experiments there was no difference in the phenotypes observed across a 100-fold range, even if there was a reduction in the magnitude of the T cell responses. From these preliminary experiments, the above protocol was optimized to balance between the volume of blood (i.e. cell numbers), and the numbers and sizes of the peptide pools.

### *Ex vivo* stimulation

PBMC were rapidly thawed in RPMI 1640 (Cellgro, Manassas, VA), supplemented with penicillin (50 IU/ml)-streptomycin (50 µg/ml) (GIBCO, Carlsbad, CA), 10 µg/ml DNase (Sigma- Aldrich, St. Louis, MO) and 8% FBS (assay medium). Cells were centrifuged and resuspended in RPMI 1640, supplemented with penicillin (50 IU/ml)-streptomycin (50 µg/ml), and 8% FBS and rested overnight in a 37°C 5% CO_2_ incubator. On the day of the assay, cell viability was tested by trypan blue exclusion dye, and 1–2×10^6^ cells/well in assay medium were plated into a 96-well V-bottom plate (BD, Franklin Lakes, NJ). A 200 µl PBMC suspension was stimulated with 0.3% DMSO (no antigen control), groups of influenza peptides, Tetanus peptides, and staphylococcal enterotoxin-B (1 µg/ml, SEB, Sigma- Aldrich, St. Louis, MO) for a total of 10 hr. 10 µg/ml Brefeldin A (BD, Franklin Lakes, NJ) and 2 µM monensin (Sigma-Aldrich, St. Louis, MO) were added for the last 8 hours of culture. Anti-CD28 (4 µg/ml, Biolegend, San Diego, CA) was added with the stimulation conditions in Study 2. Both the length of the peptides, and the culture times were optimized for detection of CD4 T cell responses. Even though some anti-peptide CD8 T cell responses could be detected, the methods were not optimized for CD8 T cells and so only the CD4 T cell results are presented.

### Intracellular Cytokine Staining using 15-color flow cytometry

Antibodies used for Intracellular Cytokine Staining (ICS) are shown in Tables S1 and S2. Cells were labeled with surface antibodies then fixed and permeabilized for ICS using the micromethod [32]. Cells were acquired using a LSRII (BD Immunocytometry Systems) and analyzed using FlowJo software (Treestar, San Carlos, CA).

### Statistics

#### Study 1

Ratios of the number of cells producing distinct cytokine expression patterns (Thpp-like IFNγ^−^IL-2^+^TNFα^+^ versus Th1-like IFNγ^+^TNFα^+^) were analyzed by Wilcoxon signed-rank test to investigate the between-antigen cytokine expression differences. Ratios were analyzed to correct for changes in the overall magnitude of the responses against different peptide pools, thus focusing on the quality of the responses to each peptide pool. The paired t test was also applied on log-transformed ratios of the number of cells producing distinct cytokine expression patterns for consistency of conclusions. Raw two-sided p values were reported, as the number of multiple outcomes was not predictable.

#### Study 2

Comparisons of the number of TNFα, IFNγ, and IL-2 producing cells elicited following antigenic stimulation between subjects from pre-2009 and post-pandemic infection were performed using the Wilcoxon Rank Sum test followed by two-sample t test on log-transformed data for consistency of conclusions. Ratios of the number of cells producing distinct cytokine expression patterns were analyzed by Wilcoxon signed-rank test to investigate the between-antigen cytokine expression differences and confirmed by paired t test on log-transformed ratios of the number of cells producing distinct cytokine expression patterns. Specific predictions were made from the interpretation of data from Study 1 and one-sided p values would be appropriate to report for this study [33]. However, in order to interpret the data conservatively, raw two-sided p values are reported for both studies. The relationship between peptide analyses by different amino acid substitution matrices was determined by Spearman correlation analysis.

## Results

### Detection of qualitatively different CD4 T cell functional profiles

**Figure 1:**
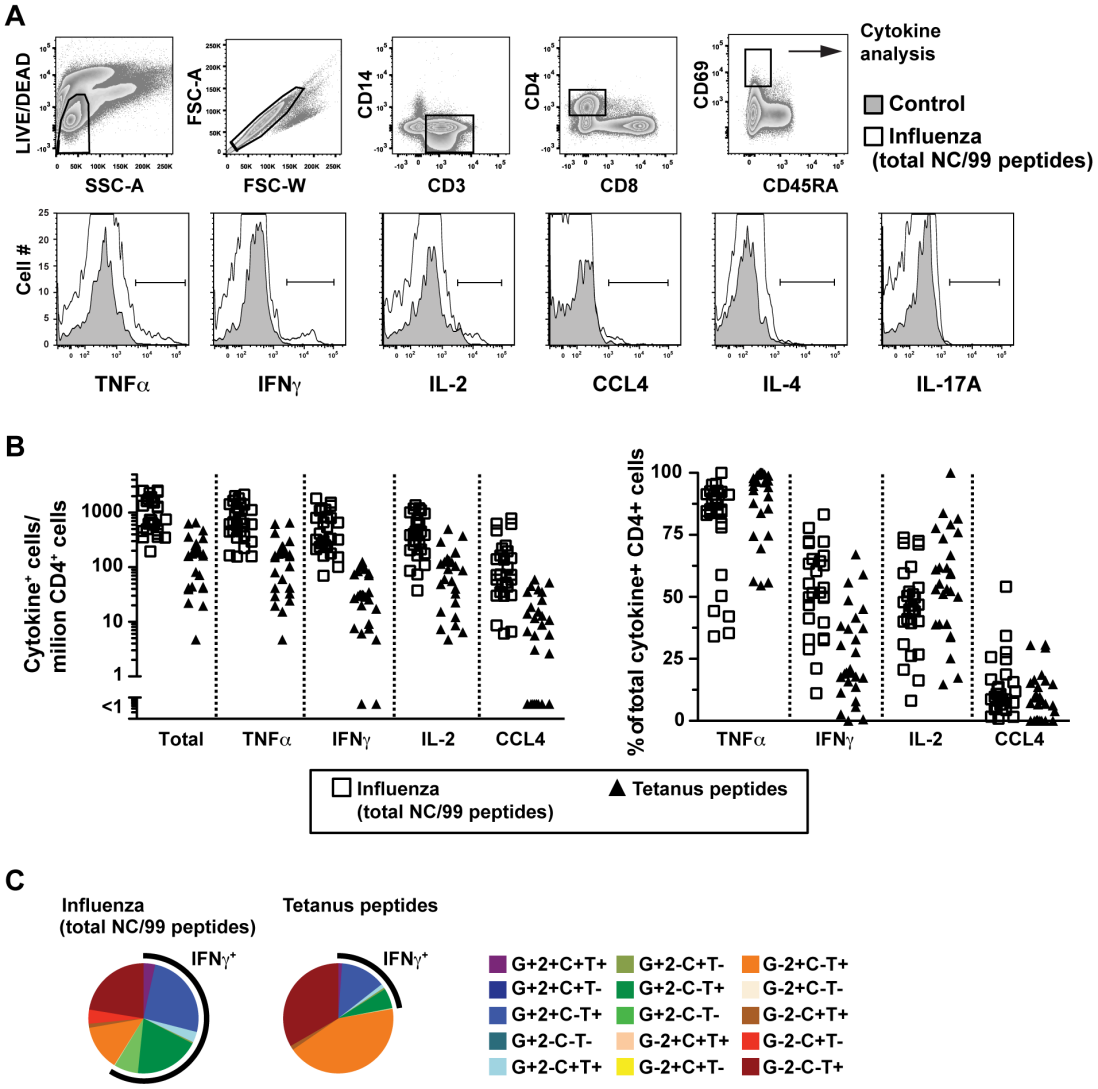
Qualitatively different memory CD4 T cell responses to influenza or tetanus. PBMC were stimulated with pools of all influenza A/New Caledonia/99 peptides, tetanus toxoid peptides or SEB (data not shown), stained for surface and intracellular markers, and analyzed by flow cytometry. (A) The gating strategy to isolate activated CD4 T cells is shown, and within this population expression of cytokines was evaluated by histogram gates. For rare cytokines such as IL-4 or IL-17A, SEB-stimulated samples were used (data not shown). (B, left) The total influenza-specific responses (cells positive for all combinations of cytokine/chemokine) and individual TNFα, IFNγ, IL-2, and CCL4 responses were calculated as numbers per million total CD4 T cells after background subtraction. (B, right) The percent of the cytokine-producing cells was calculated from the numbers in B, left. (C) Mean frequencies from all subjects for each of the cytokine positive combinations from Boolean analysis of cells secreting TNFα (T), IFNγ (G), IL-2 (2) and CCL4 (C) for influenza (left) or tetanus peptides (right). The black arcs represent the proportion of the cytokine combinations that express IFNγ.

We first evaluated the cytokine expression patterns of antigen-specific CD4 T cell responses to influenza and tetanus in a cohort of healthy adults (Table 1) by ICS and 15-color flow cytometry (Figure 1A). All subjects responded above background levels to peptides of a seasonal influenza strain that circulated for several years (A/New Caledonia/99), and most responded to tetanus peptides, at lower levels (Figure 1B, C). As expected from previous results [5], [24], [34], [35] the influenza responses were dominated by IFNγ^+^TNFα^+^ cells (mean of 58% of all cytokine-secreting cells, including variable expression of IL-2 and CCL4), whereas the tetanus responses included relatively more IFNγ^−^TNFα^+^ cells (mean of 78%, Figure 1C, D). As reported previously [6], [23], [24], the mean fluorescence intensities (MFI) of TNFα and IFNγ were higher in cells co-expressing larger numbers of cytokines (data not shown). Overall, the memory T cell response to a prevalent seasonal influenza was a polarized Th1 response, whereas the response to tetanus included a higher proportion of cells with a Thpp-like phenotype (IFNγ^−^IL-2^+^TNFα^+^).

**Table 1:** Normal Donor Patient demographics.

Pt ID	Age	Gender	Draw Date	HAI titer*
1	47	M	07/2009	10
2	48	F	07/2009	<10
3	41	F	07/2009	<10
4	49	F	07/2009	<10
5	33	F	07/2009	<10
6	49	F	08/2009	<10
7	49	F	08/2009	<10
8	25	F	08/2009	10
9	20	F	08/2009	40
10	20	F	12/2009	<10
11	20	M	04/2010	40
12	30	M	04/2010	320 #
13	39	F	04/2010	40 #
14	22	F	04/2010	160 #
15	18	F	04/2010	640
16	30	F	05/2010	80
17	21	F	05/2010	<10
18	31	F	06/2010	40
19	47	F	07/2010	20
20	19	F	07/2010	10
21	20	M	07/2010	40
22	41	M	08/2010	10
23	24	F	08/2010	20 #
24	41	F	08/2010	10
25	33	M	08/2010	40
26	37	F	10/2010	640 #
27	40	F	10/2010	40

* HAI titer to the HA derived from A/California/04/09.

# pandemic H1N1 vaccine received prior to time of blood draw.

### Temporally distinct influenza viruses to assess recent and long-term memory T cell responses

**Figure 2:**
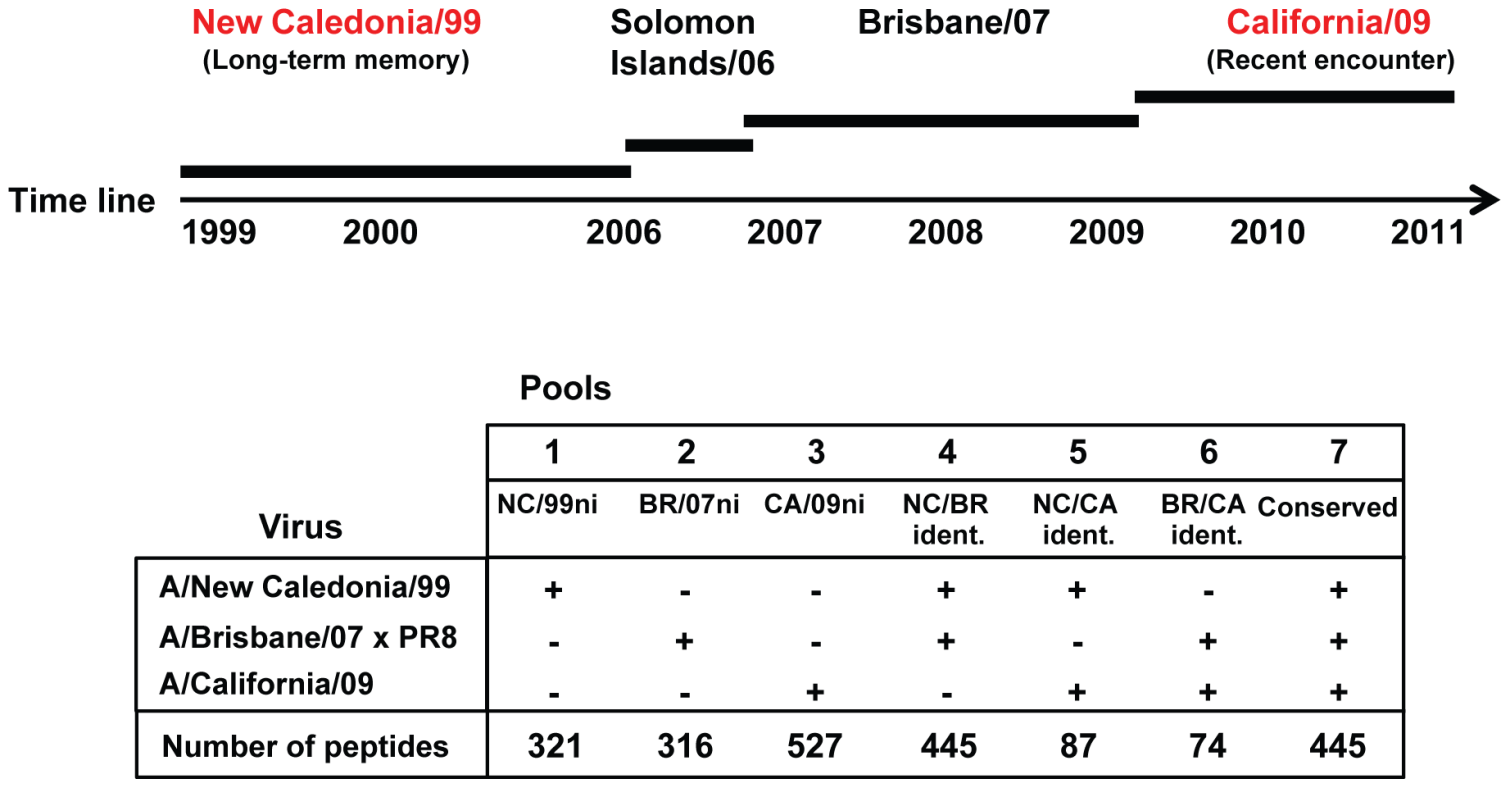
Generation of influenza-specific peptide pools reflecting antigen history in the general population. Peptide libraries were generated from the protein sequences of New Caledonia/99, Brisbane/07, and California/09 viruses that were most prevalent at different times over the past decade. The amino acid sequences of each peptide were compared across virus strains and denoted as identical (+) or non-identical (−).

To examine the phenotypes of H1N1 influenza-specific T cells with different histories of antigen stimulation, we generated pools of peptides that were selectively expressed in three temporally distinct H1N1 viruses: A/New Caledonia/20/99 (NC/99, prevalent from 1999 to 2006 and the H1N1 component of vaccines from 2000 to 2006); A/Brisbane/59/07×PR8 (BR/07) in vaccines from 2008–2010); and A/California/04/09 (CA/09, the 2009 pandemic H1N1 influenza strain). In the initial study, peptides were classified as ‘identical’ (no amino acid changes) or ‘non-identical’ across the three virus strains [36], resulting in seven different peptide groups (Figure 2). Although some ‘non-identical’ peptides with minimal changes probably contained cross-reactive epitopes, the non-identical pools should have been enriched for non-cross-reacting epitopes. We therefore expected the following trends in the responses to four distinct classes of peptide specificities: 1) NC/99 non-identical (NC/99ni, Pool 1, Figure 2) peptides should be more likely to stimulate long-term, resting memory responses; 2) BR/07 non-identical (BR/07ni, Pool 2, Figure 2) peptides should stimulate long-term, resting and recently-stimulated memory responses; 3) Peptides shared between one or more strains (pools 4, 5, 6, 7, Figure 2) should preferentially stimulate multiply-boosted responses; and 4) CA/09 non-identical (CA/09ni, Pool 3, Figure 2) should preferentially stimulate recent or primary responses.

### Increased IFNγ^−^IL-2^+^ CD4 T cell patterns in recent versus long-term memory to influenza

**Figure 3:**
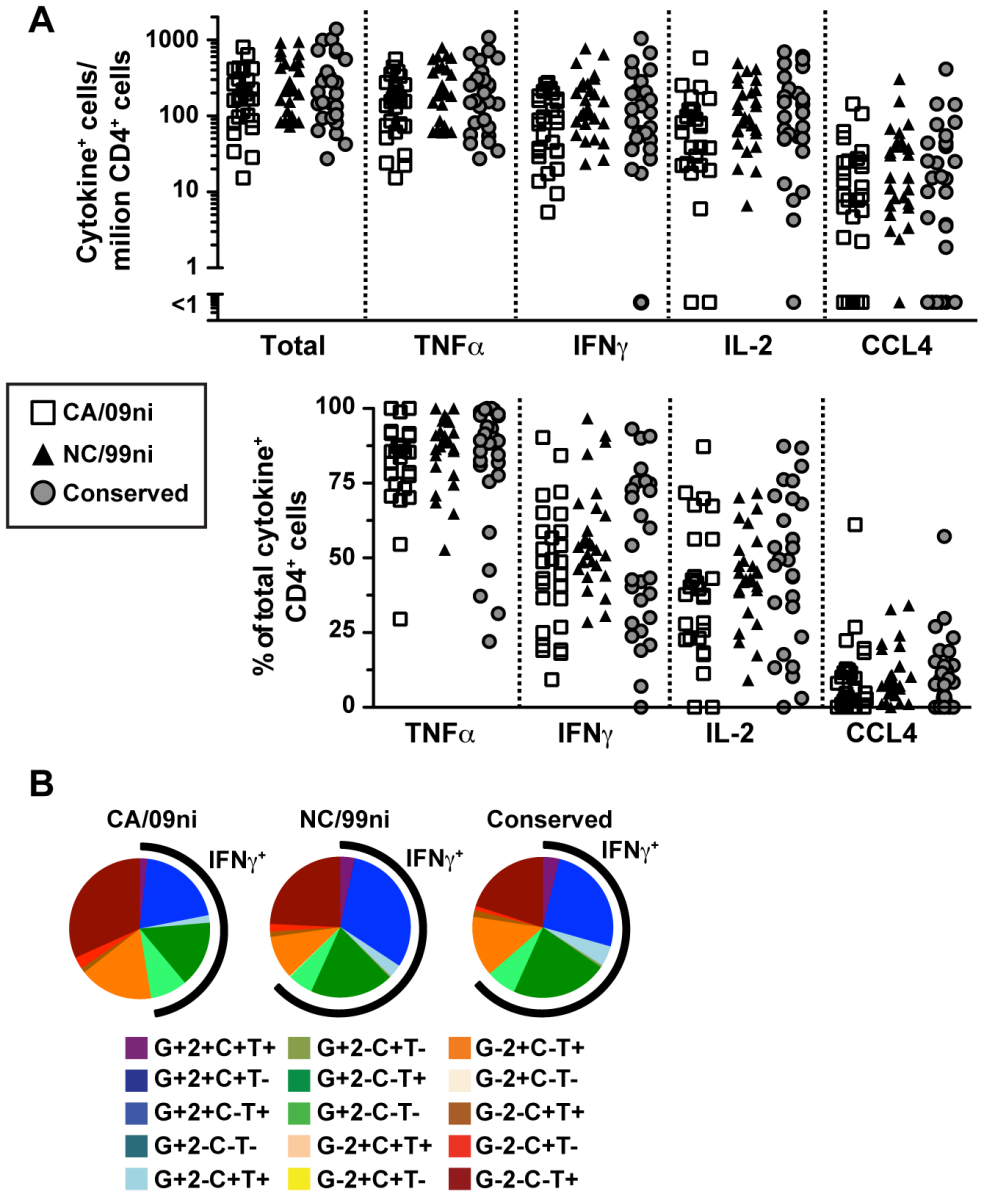
Cytokine patterns of memory CD4 T cells specific for distinct influenza specificities. PBMC were stimulated with pools of CA/09ni, NC/99ni, and conserved peptides and analyzed by ICS and flow cytometry. (A, top) The total influenza-specific responses (cells positive for all combinations of cytokine/chemokine) and individual TNFα, IFNγ, IL-2, and CCL4 responses were calculated as numbers per million total CD4 T cells after background subtraction. (A, bottom) The percent of the cytokine-producing cells was calculated from the numbers in A, top. (B) Mean frequencies from all subjects for each of the cytokine positive combinations were calculated from a Boolean analysis of cells secreting TNFα (T), IFNγ (G), IL-2 (2) and CCL4 (C) for CA/09ni (left), NC/99ni (middle), and conserved (right) peptides. The black arcs represent the proportion of the cytokine combinations that express IFNγ.

Within the overall Th1-polarized influenza response, a single antigen exposure may result in a mixed Th1 and Thpp response even to viral antigens, as Th1 differentiation may require several cycles of stimulation to become completely committed and stabilized [37]. The quality of the CD4 T cell response was compared between recently primed memory (CA/09ni peptides), and long-term quiescent memory that was unlikely to have been boosted by infection or vaccination in recent years (NC/99ni peptides), using the pools of peptides described above. In the initial study of normal adult subjects (Table 1), blood samples were taken after the H1N1 pandemic reached Rochester, NY in June 2009, and the subjects were therefore variably exposed to the H1N1 virus. All subjects responded to at least some pools of influenza peptides (Figure 1 and data not shown), and most responded to CA/09ni, NC/99ni and conserved pools (Figure 3A). Although all three peptide pools induced responses that included TNFα, IFNγ, IL-2, and CCL4, a Boolean analysis of the expression of these cytokines (Figure 3B) showed that the mean proportion of IFNγ^+^ cells (black arc) was higher in the NC/99ni and conserved peptide responses, whereas the IFNγ^−^ cells contributed more strongly to the CA/09ni responses. However, this trend in the proportion of different phenotypes was not statistically significant for the unpaired results, due to the considerable variation in response between subjects (Figure 3A).

**Figure 4:**
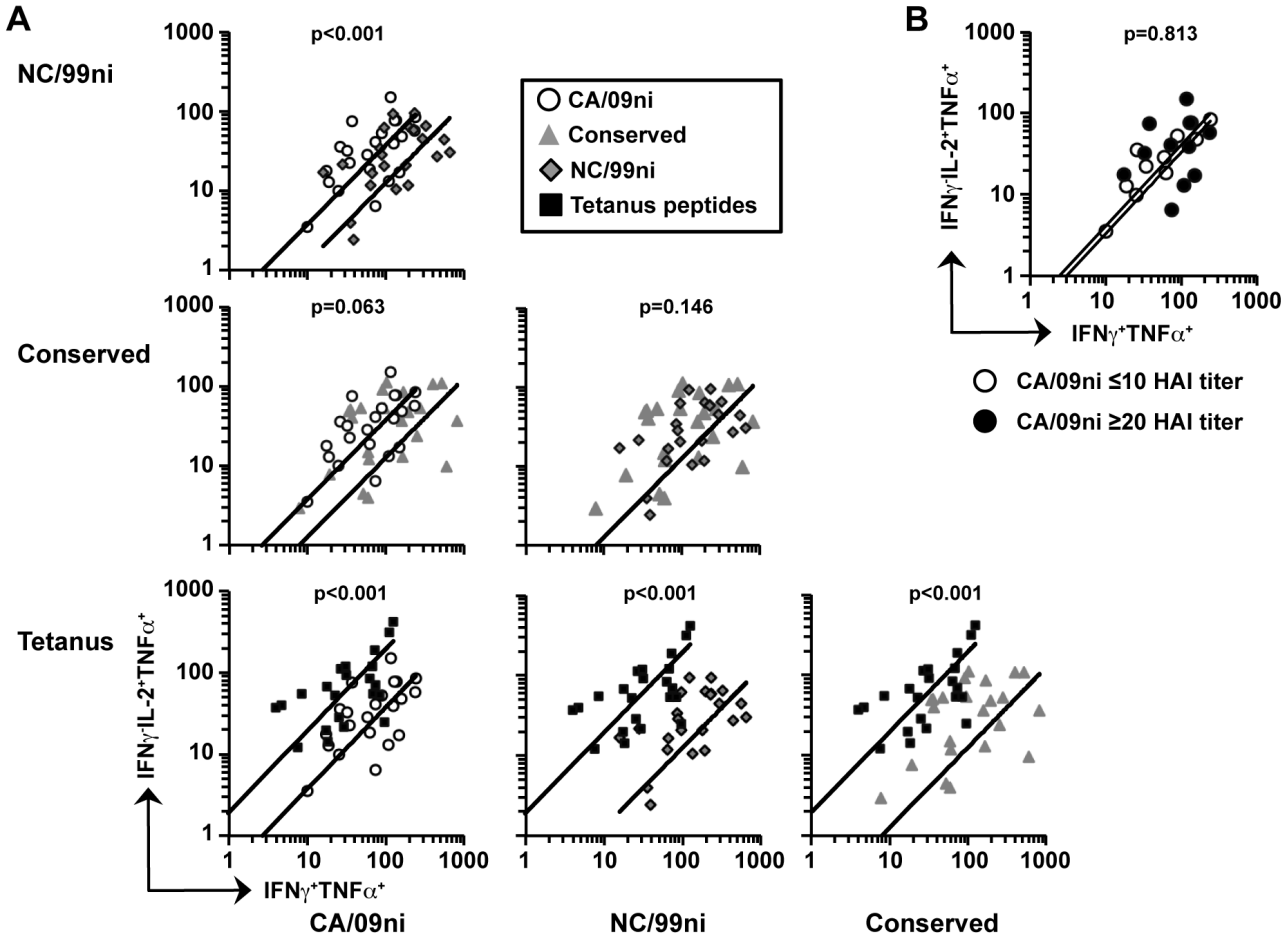
CA/09ni-specific CD4 T cells include a higher proportion of IFNγ^−^IL-2^+^ cells. The samples described in Figure 3 were analyzed by plotting the numbers of IFNγ^−^IL-2^+^TNFα^+^ versus IFNγ^+^IL-2^+^ cells within each subject, with trend lines. (A) Each plot compares two different antigen stimulations and the numbers per million total CD4 T cells are shown after background subtraction. (B) The responses to CA/09ni are compared for subjects with an anti-A/California/09 HAI antibody titer ≤10 (open) or ≥20 (closed). Two-sided, raw p-values are reported above each condition.

The trend in increased proportion of IFNγ^−^TNFα^+^ cells in the CA/09 response was further examined in a paired analysis comparing IFNγ^+^TNFα^+^ cells (Th1-like cells) with IFNγ^−^IL-2^+^TNFα^+^ cells (Thpp-like cells) in individual subjects. Comparisons between the tetanus, NC99ni, conserved, and CA09ni peptide pool responses are shown in Figure 4. The tetanus CD4 T cell response was polarized towards a Thpp-like phenotype in comparison to each of the three influenza responses (Figure 4A, bottom row), but particularly the NC/99ni and conserved responses. The CA/09ni-specific CD4 T cell response was also polarized towards IFNγ^−^IL-2^+^TNFα^+^ cells in comparison to the NC/99ni-specific response (p<0.0001), the conserved response (p = 0.063), or the sum of the NC/99ni+conserved response (p<0.0001, data not shown). Thus the recent memory responses to CA/09ni were biased towards a Thpp-like phenotype whereas the long-term quiescent responses to NC/99ni, and multiply boosted responses to conserved peptides, were more Th1-like.

The IL-2^+^ producing T cell bias was not due to overlap of the IL-2^+^ T cells with T follicular helper (Tfh) cells [15], [38], as the majority of both IFNγ^+^TNFα^+^ and IFNγ^−^IL-2^+^TNFα^+^ influenza-specific cells responding to CA/09 and NC/99 were found within the CXCR5^−^ CD4 T cell population (Figure S1). Fourteen of the 29 subjects had a detectable antibody titer (≥1∶20) to the hemagglutinin of A/California/04/09 at the time of blood draw (Table 1), but these titers did not correlate with a bias toward IL-2 production (Figure 4B).

### Strategy for detection of memory CD4 T cells induced by recent pandemic H1N1 infection

To improve the focus of our T cell analysis on the differences in new memory responses to the CA/09 influenza virus, a second clinical study was performed. Samples collected from normal donors before 6/2009 (therefore unexposed to the CA/09 virus) were compared with samples collected from a second set of donors 28 days after PCR-confirmed infection with CA/09 (Table 2).

**Table 2:** Pre-2009 and post-pandemic infection subject demographics.

Pt ID	Age	Gender	Draw Date
**Pre-2009**			
1	25	M	10/22/08
2	62	F	10/22/08
3	44	F	10/28/08
4	54	F	10/28/08
5	58	F	10/28/08
6	42	F	10/29/08
7	58	F	10/29/08
8	22	F	10/30/08
9	26	F	10/30/08
10	29	M	03/08/11
**Infection**			
1	34	F	11/09/12
2	29	M	12/15/09
3	47	F	10/09/12
4	44	M	11/09/12
5	31	M	12/15/09
6	44	F	12/15/09
7	48	F	12/22/09
8	52	F	12/17/09

The peptide pools were also modified to increase the resolution of new anti-CA/09 responses. The CA/09 non-identical peptides were ranked according to the more conservative scores (relative to NC/99 and BR/07 protein sequences) derived from two amino acid substitution matrices [39], [40], and the rank scores were averaged between the two comparison matrices. The values from the two matrices were significantly correlated (Spearman r = 0.9146, p<0.0001, Figure S2). The most-different half of the peptides were pooled as the ‘very different’ pool (vdiff) and the less-different half as the ‘different’ (diff) pool (Table S3 and Figure S2). The CA/09ni vdiff peptide pool was predicted to be further enriched for non-cross-reactive peptides, and responses to this pool were expected to be mostly due to primary responses against CA/09 infection.

Two additional pools were produced containing peptides present in one or more previously circulating influenza viruses. One pool was produced from the CA/09/NC/99 identical and CA/09/BR/07 identical peptides (Figure 2, pools 5+6), and a second pool from the NC/99/BR/07 identical and conserved peptides (Figure 2, pools 4+7). These two pools of peptides should preferentially stimulate memory T cell responses that had been multiply boosted via both vaccination and infection.

### Recent infection induced a strong increase in CD4 T cell responses

**Figure 5:**
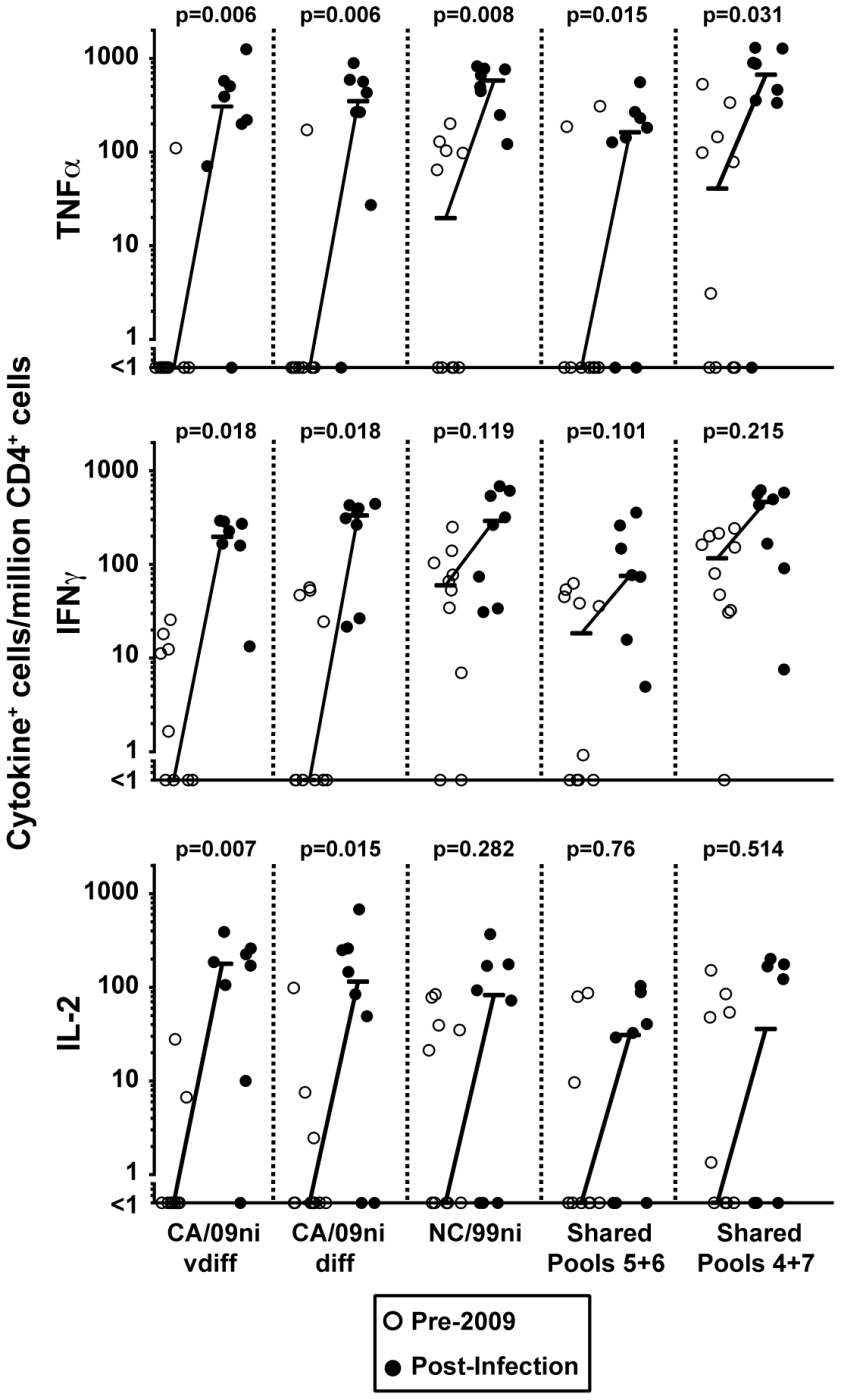
Infection with pandemic A/California/09 enhances CD4 T cell responses to shared and unique influenza antigens. PBMC samples taken before the A/California/09 pandemic (Pre-2009) or 28 days after PCR-confirmed infection (Post-Infection) were analyzed by stimulation with influenza peptide pools, ICS and flow cytometry. Numbers of influenza-specific CD4 T cells expressing TNFα, IFNγ, and IL-2 are represented as numbers per million total CD4 T cells after background subtraction. The median values of the cells expressing TNFα, IFNγ, or IL-2 were plotted with lines connecting the Pre-2009 and Post-Infection group values. Two-sided, raw p-values are reported above each condition.

As expected, samples collected prior to the 2009 pandemic H1N1 responded robustly to NC/BR identical/conserved and NC/99ni pools of peptides, whereas there was little to no immunity detected against either CA/09ni vdiff or CA/09ni diff peptide pools (Figure 5). One month after confirmed CA/09 infection, TNFα, IFNγ, and IL-2 responses against all peptide pools were higher in comparison to the unexposed cohort, and most of these increases were significant (Figure 5). Responses against CA/09ni vdiff and diff were detectable in most samples taken after confirmed infection. The fold increase in cytokine responses was generally higher in the CA/09 responses; for example the median IFNγ^+^ response against CA/09ni vdiff peptides was ∼200-fold higher in the infected versus pre-2009 group, whereas the response against NC/99ni peptides was ∼5-fold higher after infection. The NC/99ni peptide pool included all peptides with even one amino acid difference from A/California/09, and so the moderate increase in this response was probably derived mainly from cross-reactive responses.

### Responses to recent CA/09 infection were biased towards a Thpp-like phenotype

**Figure 6:**
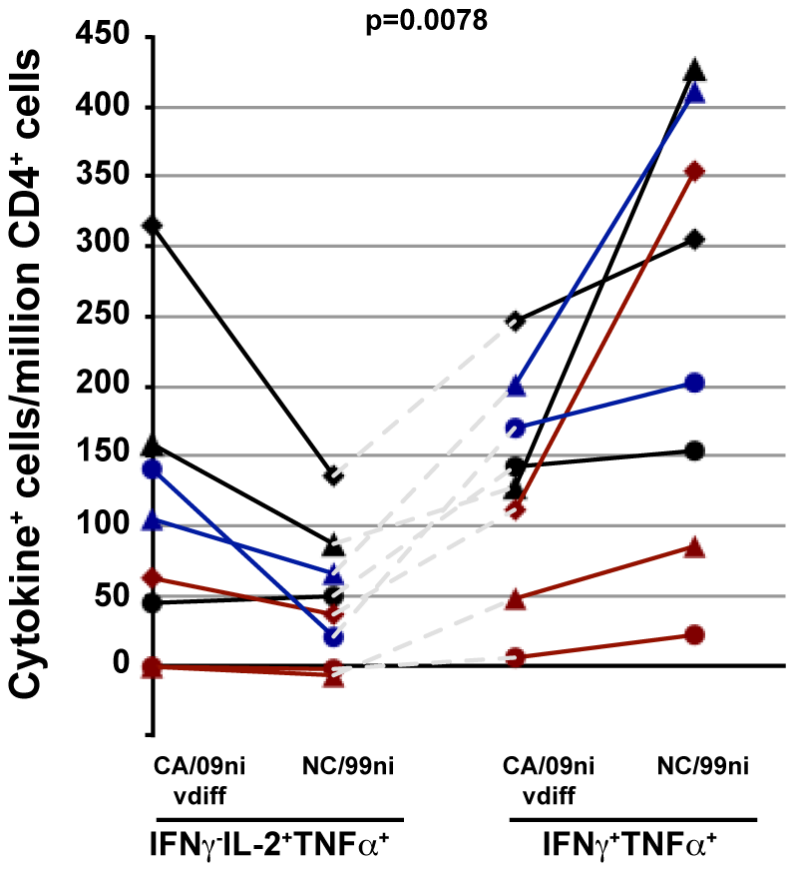
Recent infection with pandemic H1N1 preferentially induces IFNγ^−^IL-2^+^TNFα^+^ cells. The samples from infected subjects described in Figure 5 were analyzed for relative expression of different cytokine phenotypes by plotting the numbers of IFNγ^−^IL-2^+^TNFα^+^ cells (left) or IFNγ^+^TNFα^+^ cells (right) for CA/09ni vdiff and NC/99ni. The lines connect the responses to individual subjects. Color-coding is for visualizing individual subjects. The two-sided, raw p-value is reported for the differences in the ratio of the cell numbers of IFNγ^−^IL-2^+^TNFα^+^ cells versus IFNγ^+^TNFα^+^ cells elicited after CA/09ni vdiff and NC/99ni stimulation within each subject.

Based on the results of the first study showing a selective increase in Thpp-like responses against CA/09ni peptides, the Thpp-like (IFNγ^−^IL-2^+^TNFα^+^) and Th1-like (IFNγ^+^TNFα^+^) cytokine responses were compared in the recently infected subjects. Stimulation with NC/99ni or CA/09ni vdiff peptides typically induced responses above the no antigen control (Figure 6). The NC/99ni induced strong Th1-like responses, as observed in the first study (Figures 1 and 3). In contrast to these responses, the CA/09ni vdiff pool, expected to include mainly non-cross-reactive CA/09-specific peptides, induced a response biased significantly to the Thpp-like IFNγ^−^IL-2^+^TNFα^+^ phenotype, consistent with the results of the initial study. This difference was confirmed by a paired analysis linking, for each subject, the phenotypic responses to CA/09ni vdiff and NC99ni peptides for IFNγ^−^IL-2^+^TNFα^+^ (Figure 6, left), or CA/09ni vdiff and NC99ni peptides for IFNγ^+^TNFα^+^ (Figure 6, right).

## Discussion

This study took advantage of the sequences of influenza viruses circulating at different times, to compare the CD4 T cell responses generated by different antigen exposure histories. Most people have encountered influenza at a very early age [41], [42], and it is a rarity to find an adult without an anti-influenza response [43]. However, some peptides unique to the recent pandemic CA/09 influenza virus would be expected to not cross-react to previous strains. Our peptide pool strategy used information from different H1N1 influenza strains to selectively stimulate T cells specific for epitopes on viruses circulating for a long time, and to contrast these responses with those against epitopes unique to the recent pandemic CA/09 influenza – these latter responses should be at least partially enriched for primary responses. In both the cross-sectional study and the recent-infection study, qualitatively different responses, i.e. increased numbers of IFNγ^−^IL-2^+^TNFα^+^ cells, occurred against the peptides from the recent influenza CA/09. Thus there may be a difference in the quality of recently induced memory CD4 T cells in comparison to memory cells that have persisted over many years and/or may have been multiply boosted.

Despite major advances in predictive algorithms, such as using artificial neural networks or consensus predictions, there are currently no algorithms available that accurately predict the immunogenicity of a peptide across the multitude of HLA class II alleles [44], [45]. MHC alleles of the subjects in our studies were not determined, but probably included normal diversity. Therefore a comparative analysis of the possible epitopes within the various peptide pools was not feasible. Accordingly, we initially used a one amino acid difference cutoff to assess T cell function independent of the contributions from HLA type and TcR so that we could identify virus-specific responses within the general population. This method is inclusive [46], [47] and should identify many peptides identical between strains. Although some peptides with only slight differences, e.g. one amino acid, may still cross-react antigenically, this method should also provide partial enrichment for non-cross-reactive epitopes for each individual strain of influenza.

In the first (normal donor) study, the responses to conserved (identical) peptide pools should comprise a mixture of long-term memory and recently-boosted memory, depending on the individual infection history. NC/99ni responses should include the same T cell phenotypes, possibly with a bias towards more resting long-term memory because some T cells would recognize epitopes that do not cross-react with recent influenza strains. In contrast, responses to the CA/09ni peptide pool should be a mixture of recently-boosted memory (specific for non-identical but cross-reactive epitopes), and recent primary responses against new epitopes in subjects exposed to or vaccinated with the CA/09 virus. This therefore suggests that the trend towards more IFNγ^−^IL-2^+^ cells in the responses to the CA/09ni pool may be due to recent primary responses.

In the second (infection) study, the most divergent (potentially least cross-reactive) peptides of the pandemic H1N1 were identified by ranking, based upon amino acid substitution matrices that take into account the occurrence of pair-wise substitutions and physicochemical properties of each amino acid [39], [40]. Soon after pandemic H1N1 infection, responses were higher against all peptide pools, even the NC/99ni pool, suggesting that cross-reaction between NC/99 and CA/09 peptides did occur in this pool. The increased NC/99ni and memory flu peptide responses maintained the bias toward IFNγ^+^TNFα^+^ producing cells seen in the healthy donor study, and the pre-pandemic blood samples. The CA/09ni diff peptides may also have contained cross-reactive peptides, as this pool also induced Th1-biased responses (data not shown). However, the CA/09ni vdiff pool, representing the peptides least likely to cross-react with epitopes in previous influenza strains, revealed a strongly enhanced response biased towards IFNγ^−^IL-2^+^TNFα^+^ CD4 T cells after PCR-confirmed CA/09 infection.

The investigation of naïve and recently infected subjects by enrichment of virus-specific sequences and 15-color flow cytometry is an effective method for rapid analysis of an emerging pathogen, as only the protein sequences from a representative isolate are required to generate peptide libraries, and this method identifies the relative quantity and quality of preexisting or newly-induced cell-mediated immunity. The validity of this approach is supported by two sets of results – first, recent infection induced larger expansion of the responses to two pools of CA/09ni peptides than the responses to conserved peptides, and second, the response to the CA/09ni vdiff pool was qualitatively different.

In mice, repeated stimulation of CD4 T cells reduced the percentage of cells secreting effector cytokines *in vitro* [48], and this was associated with reduced protection from influenza challenge. Also in mice, differential numbers and functional phenotypes of memory CD8 T cells were generated by heterologous prime-boost regimens differing in the boosting vector, antigen load, and level of inflammation [49]–[51]. Interestingly, a higher proportion of IFNγ^+^TNFα^+^ CD8 T cells were maintained following multiple rounds of stimulation, with a gradual loss of IL-2 production [49], [50] and changes in the regulation of additional genes. Consistent with these mouse studies, our human data also suggest that the T cell response can further evolve with repeated stimulation.

The difference between the response to the recent CA/09 epitopes and long-term memory may be due to the difference between long-term, multiply-boosted responses versus primary responses, as suggested above. A second possibility is that recent responses have a higher representation of IFNγ^−^IL-2^+^TNFα^+^ cells than long-term resting memory, because these cells are reduced in the circulation over time, due to shorter half-lives, conversion, or distribution to tissues. A third possibility is that A/California/09 may generate a different type of immune response than the previously circulating H1N1 strains. Differences in the NS1 protein may alter the production of type 1 interferons and hence other anti-viral genes [52]–[54], and changes in the hemagglutinin receptor-binding domain may alter cellular tropism and infectivity [55], potentially modifying antigen capture and functions of dendritic cells, and altering T cell differentiation.

What is the phenotype of the IFNγ^−^IL-2^+^TNFα^+^ cells induced by primary CA/09 immunization? These cells may be similar to the IFNγ^−^IL-2^+^ Thpp cells that we have described previously in mice [10], [13] and humans [5]. In addition to the distinctive cytokine profile, Thpp cells are uncommitted with respect to further acquisition of effector functions, and can differentiate into either Th1 or Th2 cells [5]. This uncommitted Thpp phenotype is consistent with the T stem cell phenotype proposed as a long-lived, self-renewing memory population [56], although the commitment state of the influenza-specific IFNγ^−^ cells has not yet been tested. These cells may also be included within the CD4 central memory (Tcm) population [14]. Expression of CD62L, often used as an identifying characteristic of Tcm cells [14], was not tested in the current study because samples were frozen before analysis, and CD62L is variably lost from the cell surface after freezing and thawing. In previous studies, Thpp cells were variable in expression of CD62L. The IFNγ^−^IL-2^+^TNFα^+^ influenza-specific cells in the present study did not express CXCR5, and so do not fit a Tfh definition [57], [58] or the more stringent Tcm definition used in one study [15].

The CD4 Tcm, CD4 stem cells and Thpp cells have been proposed to act as a reservoir of memory cells with high proliferative potential and low effector function, that can give rise during subsequent infections to effectors with stronger effector functions but more limited proliferative capacity [26], [56], [59]. Thus a response to either vaccination or infection may be most effective when balanced to include both effector memory cells that can immediately respond with strong effector functions during a subsequent infection, as well as Thpp-like cells that provide long-term expansion and preservation of the memory pool, and can differentiate into fresh effector cells.

The balance between effector memory and highly proliferative stem-cell like populations may be particularly important for influenza, as most people are exposed repeatedly to influenza infections, and many are also immunized on a yearly basis. As antibody epitopes are changed rapidly on influenza variants, cross-reactive T cells may be an important component of the response both as helpers for antibody and CTL responses, and also as effectors in their own right [60]. As different influenza vaccine strategies are pursued, including adjuvanted vaccines [61] and a pan-influenza vaccine [62], [63], it may be important to maintain a balance between effectors and the IFNγ^−^IL-2^+^TNFα^+^ producing cells, similar to that observed with tetanus toxoid and other protective protein vaccines [5].

## Supporting Information

Figure S1 **IFNγ^−^IL-2^+^TNFα^+^ T cells are not a subpopulation of CXCR5^+^ helper T cells.** Activated CD4 memory cells (CD3^+^CD14^−^CD4^+^CD8^−^CD45RA^−^CD69^+^) in the samples described in Figure 1 were gated into CXCR5^−^ (open) or CXCR5^+^ (closed) populations, and the numbers of IFNγ^+^TNFα^+^ cells were plotted against IFNγ^−^IL-2^+^TNFα^+^ cells after background subtraction.
(TIF)

Figure S2 **Enrichment of CA/09-specific peptides utilizing amino acid substitution matrices.** The amino acid sequences representing CA/09ni (Figure 2, Table S3) were further enriched using amino acid substitution matrices. Each peptide in CA/09ni (Pool 3) was compared to the protein sequences of NC/99 and BR/07 using the Immune Epitope Database's Epitope Conservancy Analysis tool to determine the individual amino acid sequence variations. (A) Each peptide was given two scores based upon BLOSUM62 and Yu amino acid substitution matrices. These scores correlated well (Spearman correlation coefficient r and p values are shown). (B) The rank scores were averaged between the two matrices, and the peptides were then divided into ‘different’ and ‘very different’ peptide pools by splitting the pool in half. The chart shows the resulting numbers of peptides having 1, 2, 3, or ≥4 amino acid differences in each protein, in the different and very different pools.
(TIF)

Table S1 **Antibody panel for cytokine staining (Study 1).**
(DOCX)

Table S2 **Antibody panel for cytokine staining (Study 2).**
(DOCX)

Table S3 **Influenza peptide pools used for selective T cell stimulation.**
(DOCX)
