# Supplementary figures and images for: Increase in IFNγ−IL-2+ Cells in Recent Human CD4 T Cell Responses to 2009 Pandemic H1N1 Influenza
Source: PLoS One. 2013 Mar 20;8(3):e57275. doi: 10.1371/journal.pone.0057275 (PMC3603952; doi:10.1371/journal.pone.0057275)

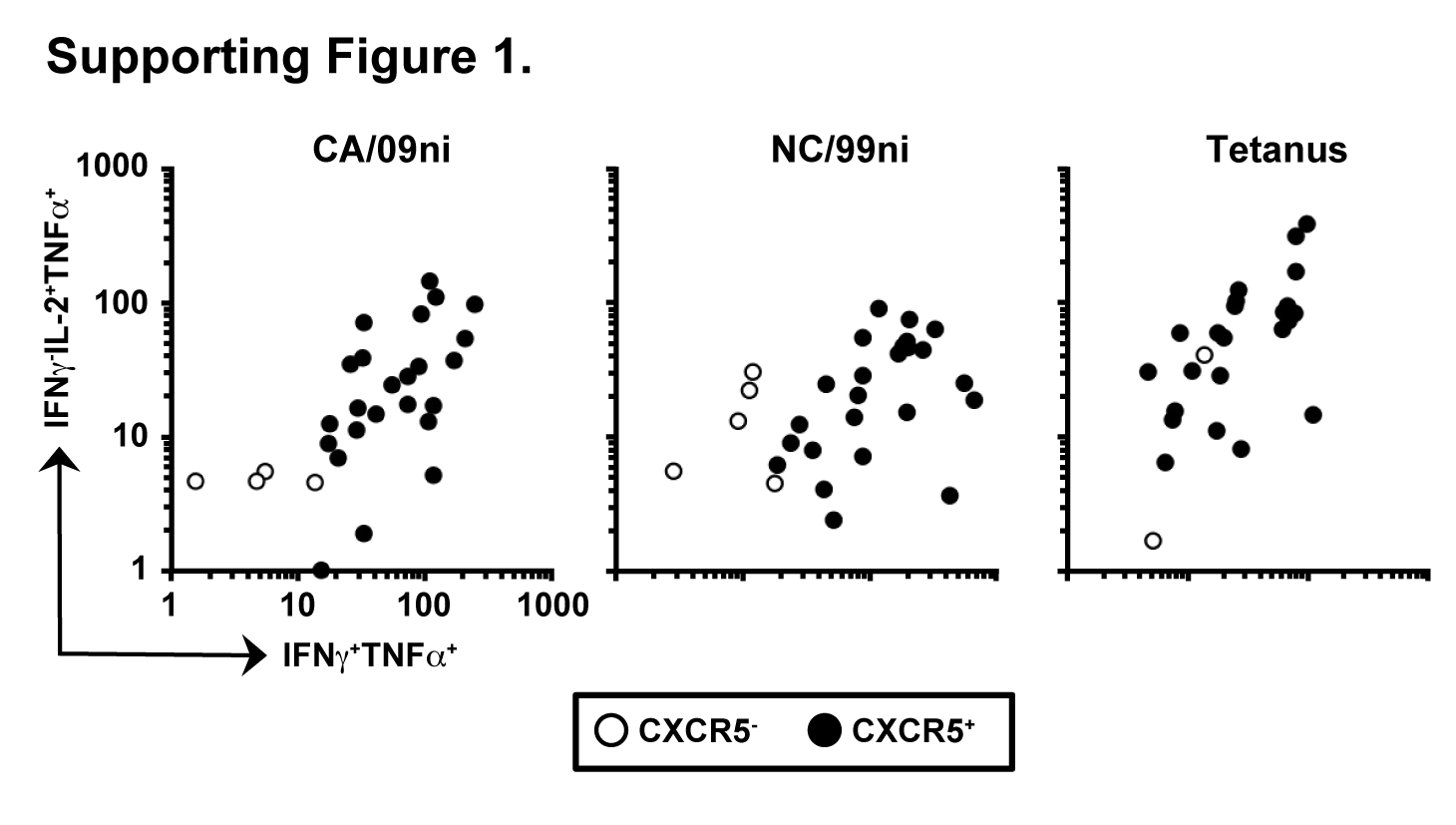

Supplement: Figure S1 — IFNγ−IL-2+TNFα+ T cells are not a subpopulation of CXCR5+ helper T cells. Activated CD4 memory cells (CD3+CD14−CD4+CD8−CD45RA−CD69+) in the samples described in Figure 1 were gated into CXCR5− (open) or CXCR5+ (closed) populations, and the numbers of IFNγ+TNFα+ cells were plotted against IFNγ−IL-2+TNFα+ cells after background subtraction. (TIF) [file pone.0057275.s001.tif]

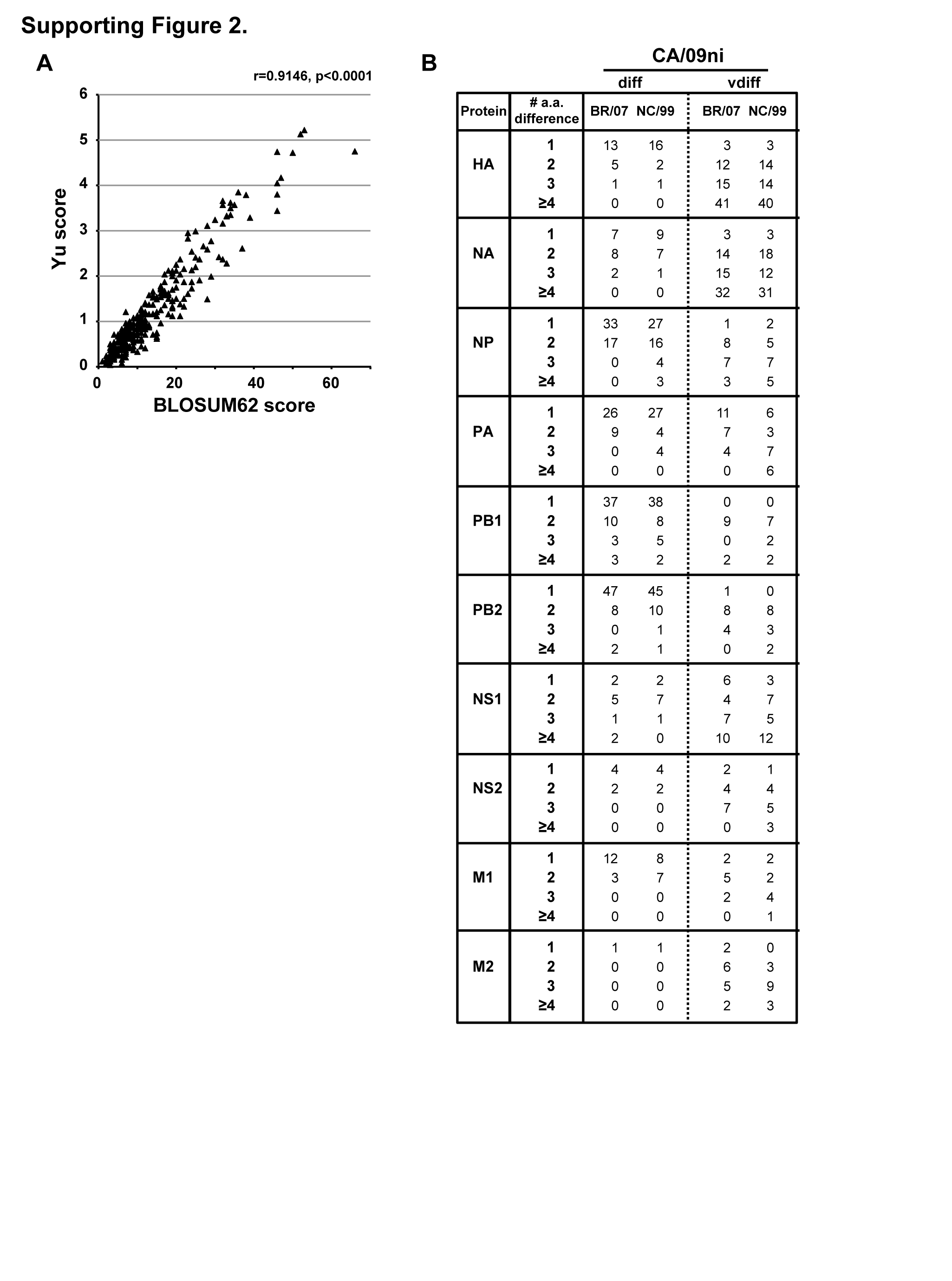

Supplement: Figure S2 — Enrichment of CA/09-specific peptides utilizing amino acid substitution matrices. The amino acid sequences representing CA/09ni (Figure 2, Table S3) were further enriched using amino acid substitution matrices. Each peptide in CA/09ni (Pool 3) was compared to the protein sequences of NC/99 and BR/07 using the Immune Epitope Database's Epitope Conservancy Analysis tool to determine the individual amino acid sequence variations. (A) Each peptide was given two scores based upon BLOSUM62 and Yu amino acid substitution matrices. These scores correlated well (Spearman correlation coefficient r and p values are shown). (B) The rank scores were averaged between the two matrices, and the peptides were then divided into ‘different’ and ‘very different’ peptide pools by splitting the pool in half. The chart shows the resulting numbers of peptides having 1, 2, 3, or ≥4 amino acid differences in each protein, in the different and very different pools. (TIF) [file pone.0057275.s002.tif]
