# Supplementary material for: Increase in IFNγ−IL-2+ Cells in Recent Human CD4 T Cell Responses to 2009 Pandemic H1N1 Influenza
Source: PLoS One. 2013 Mar 20;8(3):e57275. doi: 10.1371/journal.pone.0057275 (PMC3603952; doi:10.1371/journal.pone.0057275)
Supplement: Table S2 — Antibody panel for cytokine staining (Study 2). (DOCX) [file pone.0057275.s004.docx]

**Table S2. Antibody panel for cytokine staining (Study 2)**

**Antigen Fluorochrome Clone**

Ki-67 FITC B56

CCL4 PerCPCy5.5 D21-1351

TNFα Pacific Blue MP9-20A4

Live/Dead Yellow N/A

Granzyme B biotin-SA Qdot585 GB11

CD3 Qdot605 UCHT1

CD45RA Qdot655 MEM-56

CD8 Qdot705 3B5

CD14 Qdot800 TuK4

IL-4 APC MP4-25D2

IL-2 AF700 MQ1-17H12

CD69 APC Cy7 FN-50

GM-CSF PE BVD2-21C11

CD4 PE TR S3.5

CXCR3 PE Cy5 1C6/CXCR3

IFNγ PE Cy7 B27
