# Supplementary material for: Increase in IFNγ−IL-2+ Cells in Recent Human CD4 T Cell Responses to 2009 Pandemic H1N1 Influenza
Source: PLoS One. 2013 Mar 20;8(3):e57275. doi: 10.1371/journal.pone.0057275 (PMC3603952; doi:10.1371/journal.pone.0057275)
Supplement: Table S3 — Influenza peptide pools used for selective T cell stimulation. (DOCX) [file pone.0057275.s005.docx]

HA MKAKLLVLLCTFTATYA

HA LEKNVTVTHSVNLLEDS

HA VTHSVNLLEDSHNGKL

HA NLLEDSHNGKLCLLKGI

HA CELLISKESWSYIVETP

HA KESWSYIVETPNPENGT

HA IVETPNPENGTCYPGYF

HA PENGTCYPGYFADYEEL

HA YPGYFADYEELREQLSS

HA TVTGVSASCSHNGKSSF

HA ASCSHNGKSSFYRNLLW

HA GKSSFYRNLLWLTGKNG

HA TGKNGLYPNLSKSYVNN

HA YPNLSKSYVNNKEKEVL

HA SYVNNKEKEVLVLWGVH

HA LWGVHHPPNIGNQRALY

HA PPNIGNQRALYHTENAY

HA NQRALYHTENAYVSVVS

HA HTENAYVSVVSSHYSRR

HA VSVVSSHYSRRFTPEIA

HA HYSRRFTPEIAKRPKVR

HA IIFEANGNLIAPWYAFA

HA GNLIAPWYAFALSRGFG

HA WYAFALSRGFGSGIITS

HA SRGFGSGIITSNAPMDE

HA GIITSNAPMDECDAKCQ

HA APMDECDAKCQTPQGAI

HA LERRMENLNKKVDDGFL

HA EIGNGCFEFYHKCNNE

HA CFEFYHKCNNECMESVK

HA KCNNECMESVKNGTYDY

NA ISIWASHSIQTGSQNHT

NA HSIQTGSQNHTGVCNQR

NA SQNHTGVCNQRIITYEN

NA VCNQRIITYENSTWVNH

NA YVNINNTNVVAGKDKTS

NA TNVVAGKDKTSVTLAGN

NA KDKTSVTLAGNSSLCSI

NA GAVAVLKYNGIITETIK

NA KYNGIITETIKSWKKRI

NA TETIKSWKKRILRTQES

NA WKKRILRTQESECVCVN

NA RTQESECVCVNGSCFTI

NA CVCVNGSCFTIMTDGPS

NA SCFTIMTDGPSNGAASY

NA TDGPSNGAASYKIFKIE

NA GAASYKIFKIEKGKVTK

NA NFHYEECSCYPDTGTVM

NA CSCYPDTGTVMCVCRDN

NA TGTVMCVCRDNWHGSNR

NA GYICSGVFGDNPRPKDG

NA VFGDNPRPKDGEGSCNP

NA RPKDGEGSCNPVTVDGA

NA GSCNPVTVDGADGVKGF

NA TVDGADGVKGFSYKYGN

NA GVKGFSYKYGNGVWIGR

NA YKYGNGVWIGRTKSNRL

NA KGFEMIWDPNGWTDTDS

NA WDPNGWTDTDSDFSVKQ

NA TDTDSDFSVKQDVVAIT

NA DTANWSWPDGAELPFTI

NP RQNATEIRASVGRMIGG

NP GRFYIQMCTELKLNDYE

NP MCTELKLNDYEGRLIQN

NP LNDYEGRLIQNSLTIER

NP AGKDPKKTGGPIYKRVD

NP KTGGPIYKRVDGKWVRE

NP YKRVDGKWVRELVLYDK

NP KWVRELVLYDKEEIRRI

NP VLYDKEEIRRIWRQANN

NP RQANNGDDATAGLTHIM

NP DDATAGLTHIMIWHSNL

NP LTHIMIWHSNLNDTTYQ

NP WHSNLNDTTYQRTRALV

NP DTTYQRTRALVRTGMDP

NP RSGAAGAAVKGVGTMVL

NP AAVKGVGTMVLELIRMI

NP GTMVLELIRMIKRGIND

NP RNPGNAEIEDLTFLARS

NP EIEDLTFLARSALILRG

NP GPAVASGYDFEKEGYSL

NP GYDFEKEGYSLVGVDPF

NP EGYSLVGVDPFKLLQTS

NP GVDPFKLLQTSQVYSLI

NP LLQTSQVYSLIRPNENP

NP PNENPAHKSQLVWMACN

NP HKSQLVWMACNSAAFED

NP WMACNSAAFEDLRVSSF

NP AAFEDLRVSSFIRGTRV

NP RVSSFIRGTRVLPRGKL

NP RGTRVLPRGKLSTRGVQ

NP TRGVQIASNENMDAIVS

NP ASNENMDAIVSSTLELR

NP DAIVSSTLELRSRYWAI

NP TNQQRASAGQISTQPTF

NP SAGQISTQPTFSVQRNL

NP TQPTFSVQRNLPFDKTT

NP VQRNLPFDKTTIMAAFT

NP FDKTTIMAAFTGNTEGR

NP MAAFTGNTEGRTSDMRA

NP NTEGRTSDMRAEIIKMM

NP SDMRAEIIKMMESARPE

NP IIKMMESARPEEVSFQG

NP SARPEEVSFQGRGVFEL

NP VSFQGRGVFELSDERAT

NP GVFELSDERATNPIVPS

NP DERATNPIVPSFDMSNE

PA MIVELAEKAMKEYGEDL

PA EKAMKEYGEDLKIETNK

PA INEQGESIIVEPEDPNA

PA SIIVEPEDPNALLKHRF

PA EDPNALLKHRFEIIEGR

PA GEETIEERFEITGTLRR

PA ERFEITGTLRRLADQSL

PA GTLRRLADQSLPPNFS

PA LADQSLPPNFSCIENFR

PA PPNFSCIENFRAYVDGF

PA IENFRAYVDGFEPNGYI

PA RIEPFLKTTPRPIRLPN

PA KTTPRPIRLPNGPPCFQ

PA IRLPNGPPCFQRSKFLL

PA PPCFQRSKFLLMDSLKL

PA SKFLLMDSLKLSIEDPN

PA DSLKLSIEDPNHEGEGI

PA IEDPNHEGEGIPLYDAI

PA CMRTFFGWKEPTVVKPH

PA GWKEPTVVKPHEKGINP

PA KGINPNYLLSWKQVLEE

PA YLLSWKQVLEELQDIES

PA KQVLEELQDIESEEKIP

PA LQDIESEEKIPRTKNMK

PA EEKIPRTKNMKKTSQLK

PA APEKVDFDDCKGISDLK

PA FDDCKGISDLKQYDSDE

PA ISDLKQYDSDEPELRSF

PA YDSDEPELRSFSSWIQN

PA ELRSFSSWIQNEFNKA

PA SSWIQNEFNKACELTDS

PA EFNKACELTDSIWIELD

PA ELTDSIWIELDEIGEDV

PA YCVLEIGDMLLRSAIGQ

PA GDMLLRSAIGQVSRPMF

PA SSVKEKDMTKEFFENRS

PA DMTKEFFENRSETWPIG

PA FENRSETWPIGESPKGV

PA TWPIGESPKGVEEGSIG

PA SPKGVEEGSIGKVCRTL

PA VLLNASWFNSFLTHALR

PB1 GYTMDTVNRTHQYSERG

PB1 VNRTHQYSERGRWTKNT

PB1 YSERGRWTKNTETGAPQ

PB1 WTKNTETGAPQLNPIDG

PB1 ANTIEVFRSNGLIANES

PB1 FRSNGLIANESGRLIDF

PB1 IANESGRLIDFLKDVME

PB1 RLIDFLKDVMESMDRDE

PB1 KDVMESMDRDEVEVTTH

PB1 MDRDEVEVTTHFQRKRR

PB1 EVTTHFQRKRRVRDNVT

PB1 FQRKRRVRDNVTKKMVT

PB1 VRDNVTKKMVTQRTIGK

PB1 KKMVTQRTIGKKKHKLD

PB1 RTIGKKKHKLDKRSYLI

PB1 KHKLDKRSYLIRALTLN

PB1 ENQNPRMFLAMITYITK

PB1 MFLAMITYITKNQPEWF

PB1 TYITKNQPEWFRNILSI

PB1 NQPEWFRNILSIAPIMF

PB1 RNILSIAPIMFSNKMAR

PB1 LRTQIPAEMLANIDLKY

PB1 AEMLANIDLKYFNDSTK

PB1 IDLKYFNDSTKKKIEKI

PB1 NDSTKKKIEKIRPLLID

PB1 LQSSDDFALIVNAPNYA

PB1 FALIVNAPNYAGIQAGV

PB1 APNYAGIQAGVDRFYRT

PB1 RCHRGDTQIQTRRSFEI

PB1 TQIQTRRSFEIKKLWDQ

PB1 RSFEIKKLWDQTRSKAG

PB1 KLWDQTRSKAGLLVSDG

PB1 RSKAGLLVSDGGPNLYN

PB1 NPLNPFVSHKEIESVNN

PB1 VSHKEIESVNNAVIMPA

PB1 ESVNNAVIMPAHGPAKN

PB1 VIMPAHGPAKNMEYDAV

PB1 EYDAVATTHSWVPKRNR

PB1 TTHSWVPKRNRSILNTS

PB1 RGILEDEQMYQRCCNLF

PB1 IDFESGRIKKEEFAEIM

PB1 RIKKEEFAEIMKTCSTI

PB1 FAEIMKTCSTIEDLRRQ

PB1 TCSTIEDLRRQK

PB2 IIKKYTSGRQEKNPSLR

PB2 SGRQEKNPSLRMKWMMA

PB2 NPSLRMKWMMAMKYPIT

PB2 MIPERNEQGQTLWSKVN

PB2 EQGQTLWSKVNDAGSDR

PB2 WSKVNDAGSDRVMISPL

PB2 AGSDRVMISPLAVTWWN

PB2 MISPLAVTWWNRNGPVA

PB2 VTWWNRNGPVANTIHYP

PB2 NGPVANTIHYPKIYKTY

PB2 TIHYPKIYKTYFEKVER

PB2 IYKTYFEKVERLKHGTF

PB2 SESQLTITKEKKEELQN

PB2 ITKEKKEELQNCKISPL

PB2 EELQNCKISPLMVAYML

PB2 SLLEMCHSTQIGGTRMV

PB2 HSTQIGGTRMVDILRQN

PB2 GTRMVDILRQNPTEEQA

PB2 KREEEVLTGNLQTLKLT

PB2 LTGNLQTLKLTVHEGYE

PB2 TLKLTVHEGYEEFTMVG

PB2 HEGYEEFTMVGKRATAI

PB2 FTMVGKRATAILRKATR

PB2 LIQLIVSGRDEQSIVEA

PB2 SGRDEQSIVEAIVVAMV

PB2 SIVEAIVVAMVFSQED

PB2 IVVAMVFSQEDCMVKAV

PB2 FSQEDCMVKAVRGDLNF

PB2 MVKAVRGDLNFVNRANQ

PB2 LLRHFQKDAKVLFLNWG

PB2 KDAKVLFLNWGIEHIDN

PB2 FLNWGIEHIDNVMGMIG

PB2 EHIDNVMGMIGILPDMT

PB2 LPDMTPSTEMSMRGVRV

PB2 STEMSMRGVRVSKMGVD

PB2 RGVRVSKMGVDEYSNAE

PB2 KMGVDEYSNAERVVVSI

PB2 YSNAERVVVSIDRFLRV

PB2 MWEINGPESVLINTYQW

PB2 PESVLINTYQWIIRNWE

PB2 FQQMRDVLGTFDTTQII

PB2 VLGTFDTTQIIKLLPFA

PB2 TTQIIKLLPFAAAPPKQ

PB2 APPKQSRMQFSSLTVNV

PB2 RMQFSSLTVNVRGSGMR

PB2 LTVNVRGSGMRILVRGN

PB2 LVRGNSPVFNYNKTTKR

PB2 PVFNYNKTTKRLTILGK

PB2 KTTKRLTILGKDAGTLT

PB2 TILGKDAGTLTEDPDEG

PB2 VLRGFLILGKEDRRYGP

PB2 ILGKEDRRYGPALSINE

PB2 RRYGPALSINELSNLAK

NS1 MDSHTVSSFQVDCFLWH

NS1 SSFQVDCFLWHVRKQVA

NS1 CFLWHVRKQVADQDLGD

NS1 RKQVADQDLGDAPFLDR

NS1 DQDLGDAPFLDRLRRDQ

NS1 LRRDQKSLKGRGSTLGL

NS1 SLKGRGSTLGLNIETAT

NS1 STLGLNIETATCVGKQI

NS1 IETATCVGKQIVERILK

NS1 VGKQIVERILKEESDEA

NS1 ERILKEESDEAFKMTMA

NS1 ESDEAFKMTMASALASR

NS1 KMTMASALASRYLTDMT

NS1 ALASRYLTDMTIEEMSR

NS1 LTDMTIEEMSRDWFMLM

NS1 EEMSRDWFMLMPKQKVA

NS1 WFMLMPKQKVAGPLCVR

NS1 KQKVAGPLCVRMDQAIM

NS1 PLCVRMDQAIMDKNIIL

NS1 ANFSVIFDRLETLTLLR

NS1 FDRLETLTLLRAFTEEG

NS1 LTLLRAFTEEGAIVGEI

NS1 IVGEISPLPSLPGHTNE

NS1 PLPSLPGHTNEDVKNAI

NS1 GHTNEDVKNAIGVLIGG

NS1 VKNAIGVLIGGLEWNDN

NS1 LQRFAWRSSNETGGPPF

NS1 RSSNETGGPPFTPTQKR

NS1 GGPPFTPTQKRKMAGTI

NS1 PTQKRKMAGTIRSEV

NS2 MDSHTVSSFQDILMRMS

NS2 LMRMSKMQLGSSSGDLN

NS2 MQLGSSSGDLNGMITQF

NS2 SGDLNGMITQFESLKLY

NS2 SLKLYRDSLGEAVMRLG

NS2 DSLGEAVMRLGDLHSLQ

NS2 VMRLGDLHSLQHRNGKW

NS2 LHSLQHRNGKWREQLGQ

NS2 RNGKWREQLGQKFEEIR

NS2 FEEIRWLIEEVRHKLKT

NS2 LIEEVRHKLKTTENSFE

NS2 HKLKTTENSFEQITFMQ

NS2 ENSFEQITFMQALQLLF

NS2 ITFMQALQLLFEVEQEI

NS2 LQLLFEVEQEIRTFSFQ

M1 MSLLTEVETYVLSIVPS

M1 VETYVLSIVPSGPLKAE

M1 SIVPSGPLKAEIAQRLE

M1 AQRLEDVFAGKNTDLEA

M1 NGNGDPNNMDRAVKLYR

M1 NNMDRAVKLYRKLKREI

M1 LKREITFHGAKEIALSY

M1 FHGAKEIALSYSAGALA

M1 IALSYSAGALASCMGLI

M1 CMGLIYNRMGAVTTESA

M1 NRMGAVTTESAFGLICA

M1 TTESAFGLICATCEQIA

M1 GLICATCEQIADSQHKS

M1 CEQIADSQHKSHRQMVT

M1 SQHKSHRQMVTTTNPLI

M1 VASQARQMVQAMRAIGT

M1 RQMVQAMRAIGTHPSSS

M1 MRAIGTHPSSSTGLKND

M1 HPSSSTGLKNDLLENLQ

M2 EVETPIRNEWGCRCNDS

M2 RNEWGCRCNDSSDPLVV

M2 RCNDSSDPLVVAASIIG

M2 DPLVVAASIIGIVHLIL

M2 ASIIGIVHLILWIIDRL

M2 VHLILWIIDRLFSKSIY

M2 IIDRLFSKSIYRIFKHG

M2 SKSIYRIFKHGLKRGPS

M2 IFKHGLKRGPSTEGVPE

M2 EGVPESMREEYREEQQN

M2 MREEYREEQQNAVDADD

M2 EEQQNAVDADDGHFVSI

HA MKVKLLVLLCTFTAT

HA VTVTHSVNLLENSHN

HA SVNLLENSHNGKLCL

HA ENSHNGKLCLLKGIA

HA LLISKESWSYIVEKP

HA ESWSYIVEKPNPENG

HA IVEKPNPENGTCYPG

HA NPENGTCYPGHFADY

HA TCYPGHFADYEELRE

HA HFADYEELREQLSSV

HA TGVSASCSHNGESSF

HA SCSHNGESSFYRNLL

HA GESSFYRNLLWLTGK

HA NGLYPNLSKSYANNK

HA NLSKSYANNKEKEVL

HA YANNKEKEVLVLWGV

HA VLWGVHHPPNIGIQK

HA HHPPNIGIQKALYHT

HA IGIQKALYHTENAYV

HA ENAYVSVVSSHYSRK

HA SVVSSHYSRKFTPEI

HA HYSRKFTPEIAKRPK

HA IIFEANGNLIAPRYA

HA NGNLIAPRYAFALSR

HA APRYAFALSRGFGSG

HA FALSRGFGSGIINSN

HA GFGSGIINSNAPMDK

HA IINSNAPMDKCDAKC

HA APMDKCDAKCQTPQG

HA ENLNKKVDDGFIDIW

HA KVDDGFIDIWTYNAE

HA FIDIWTYNAELLVLL

HA IGNGCFEFYHKCNDE

HA FEFYHKCNDECMESV

HA KCNDECMESVKNGTY

NA SIWASHSIQTGSQNN

NA HSIQTGSQNNTGICN

NA GSQNNTGICNQRIIT

NA TGICNQRIITYENST

NA YVNINNTNVVAGEDK

NA NTNVVAGEDKTSVTL

NA AGEDKTSVTLAGNSS

NA GAVAVLKYNGIITGT

NA LKYNGIITGTIKSWK

NA IITGTIKSWKKQILR

NA IKSWKKQILRTQESE

NA KQILRTQESECVCMN

NA TQESECVCMNGSCFT

NA CVCMNGSCFTIMTDG

NA GSCFTIMTDGPSNKA

NA IMTDGPSNKAASYKI

NA PSNKAASYKIFKIEK

NA YEECSCYPDTGIVMC

NA CYPDTGIVMCVCRDN

NA GIVMCVCRDNWHGSN

NA YICSGVFGDNPRPED

NA VFGDNPRPEDGEGSC

NA PRPEDGEGSCNPVTV

NA GEGSCNPVTVDGANG

NA NPVTVDGANGVKGFS

NA DGANGVKGFSYKYDN

NA VKGFSYKYDNGVWIG

NA YKYDNGVWIGRTKSN

NA FEMIWDPNGWTNTDS

NA DPNGWTNTDSDFSVK

NA TNTDSDFSVKQDVVA

NP NATEIRASVGKMIGG

NP RASVGKMIGGIGRFY

NP KMIGGIGRFYIQMCT

NP IQMCTELKLSDYEGR

NP ELKLSDYEGRLIQNS

NP KTGGPIYRRVNGKWM

NP IYRRVNGKWMRELIL

NP NGKWMRELILYDKEE

NP RELILYDKEEIRRIW

NP GDDATAGLTHMMIWH

NP AGLTHMMIWHSNLND

NP MMIWHSNLNDATYQR

NP AAVKGVGTMVMELVR

NP VGTMVMELVRMIKRG

NP MELVRMIKRGINDRN

NP QVRESRNPGNAEFED

NP RNPGNAEFEDLTFLA

NP AEFEDLTFLARSALI

NP YGPAVASGYDFEREG

NP ASGYDFEREGYSLVG

NP FEREGYSLVGIDPFR

NP YSLVGIDPFRLLQNS

NP IDPFRLLQNSQVYSL

NP LLQNSQVYSLIRPNE

NP MACHSAAFEDLRVLS

NP AAFEDLRVLSFIKGT

NP LRVLSFIKGTKVLPR

NP FIKGTKVLPRGKLST

NP KVLPRGKLSTRGVQI

NP RGVQIASNENMETME

NP ASNENMETMESSTLE

NP METMESSTLELRSRY

NP TNQQRASAGQISIQP

NP ASAGQISIQPTFSVQ

NP ISIQPTFSVQRNLPF

NP TFSVQRNLPFDRTTI

NP RNLPFDRTTIMAAFN

NP DRTTIMAAFNGNTEG

NP MAAFNGNTEGRTSDM

NP GNTEGRTSDMRTEII

NP RTSDMRTEIIRMMES

NP RTEIIRMMESARPED

NP RMMESARPEDVSFQG

NP ARPEDVSFQGRGVFE

NP VSFQGRGVFELSDEK

NP RGVFELSDEKAASPI

NP LSDEKAASPIVPSFD

NP AASPIVPSFDMSNEG

PA RQCFNPMIVELAEKT

PA PMIVELAEKTMKEYG

PA LAEKTMKEYGEDLKI

PA FHFINEQGESIIVEL

PA EQGESIIVELGDPNA

PA IIVELGDPNALLKHR

PA IEERFEITGTMRKLA

PA MRKLADQSLPPNFSS

PA DQSLPPNFSSLENFR

PA PNFSSLENFRAYVDG

PA RIEPFLKTTPRPLRL

PA LKTTPRPLRLPNGPP

PA RPLRLPNGPPCSQRS

PA PNGPPCSQRSKFLLM

PA CSQRSKFLLMDALKL

PA MRTFFGWKEPNVVKP

PA GWKEPNVVKPHEKGI

PA NVVKPHEKGINPNYL

PA NPNYLLSWKQVLAEL

PA LSWKQVLAELQDIEN

PA QDIENEEKIPKTKNM

PA EEKIPKTKNMKKTSQ

PA KTKNMKKTSQLKWAL

PA DVGDLKQYDSDEPEL

PA KQYDSDEPELRSLAS

PA DEPELRSLASWIQNE

PA RSLASWIQNEFNKAC

PA EHIASMRRNYFTSEV

PA MRRNYFTSEVSHCRA

PA FTSEVSHCRATEYIM

PA KWEKYCVLEIGDMLI

PA CVLEIGDMLIRSAIG

PA GDMLIRSAIGQVSRP

PA IGESPKGVEESSIGK

PA KGVEESSIGKVCRTL

PA SSIGKVCRTLLAKSV

PA WFNSFLTHALS

PB1 DTVNRTHQYSEKGRW

PB1 THQYSEKGRWTTNTE

PB1 EKGRWTTNTETGAPQ

PB1 FLKDVMESMNKEEMG

PB1 MESMNKEEMGITTHF

PB1 KEEMGITTHFQRKRR

PB1 QRKRRVRDNMTKKMI

PB1 VRDNMTKKMITQRTM

PB1 TKKMITQRTMGKKKQ

PB1 TQRTMGKKKQRLNKR

PB1 GKKKQRLNKRSYLIR

PB1 RLNKRSYLIRALTLN

PB1 VRKMMTNSQDTELSF

PB1 TNSQDTELSFTITGD

PB1 TELSFTITGDNTKWN

PB1 ENQNPRMFLAMITYM

PB1 RMFLAMITYMTRNQP

PB1 MITYMTRNQPEWFRN

PB1 TRNQPEWFRNVLSIA

PB1 EWFRNVLSIAPIMFS

PB1 VLSIAPIMFSNKMAR

PB1 SMKLRTQIPAEMLAS

PB1 EMLASIDLKYFNDST

PB1 IDLKYFNDSTRKKIE

PB1 FNDSTRKKIEKIRSL

PB1 RKKIEKIRSLLIEGT

PB1 KIRSLLIEGTASLSP

PB1 LIEGTASLSPGMMMG

PB1 SMELPSFGVSGINES

PB1 SFGVSGINESADMSI

PB1 GINESADMSIGVTVI

PB1 KNNMINNDLGIRNLH

PB1 NNDLGIRNLHIPEVC

PB1 NPLNPFVSHKEIESM

PB1 FVSHKEIESMNNAVM

PB1 EIESMNNAVMMPAHG

PB1 NNAVMMPAHGPAKNM

PB1 NRSILNTSQRGVLED

PB1 NTSQRGVLEDEQMYQ

PB1 GVLEDEQMYQRCCNL

PB1 ESGRIKKEEFTEIMK

PB1 KKEEFTEIMKICSTI

PB1 TEIMKICSTIEELRR

PB2 KNPALRMKWMMAMKY

PB2 NEQGQTLWSKMNDAG

PB2 TLWSKMNDAGSDRVM

PB2 MNDAGSDRVMVSPLA

PB2 VSPLAVTWWNRNGPI

PB2 VTWWNRNGPITNTVH

PB2 RNGPITNTVHYPKIY

PB2 TNTVHYPKIYKTYFE

PB2 YPKIYKTYFERVERL

PB2 KTYFERVERLKHGTF

PB2 RVERLKHGTFGPVHF

PB2 SQLTITKEKKEELQD

PB2 TKEKKEELQDCKISP

PB2 EELQDCKISPLMVAY

PB2 LEMCHSTQIGGIRMV

PB2 STQIGGIRMVDILRQ

PB2 GIRMVDILRQNPTEE

PB2 DAKVLFQNWGVEPID

PB2 FQNWGVEPIDNVMGM

PB2 VEPIDNVMGMIGILP

PB2 IGILPDMTPSIEMSM

PB2 DMTPSIEMSMRGVRI

PB2 IEMSMRGVRISKMGV

PB2 RGVRISKMGVDEYSS

PB2 TERVVVSIDRFLRIR

PB2 VSIDRFLRIRDQRGN

PB2 FLRIRDQRGNVLLSP

PB2 LVNTYQWIIRNWETV

PB2 QQMRDVLGTFDTAQI

PB2 VLGTFDTAQIIKLLP

PB2 DTAQIIKLLPFAAAP

PB2 PKQSRMQFSSFTVNV

PB2 MQFSSFTVNVRGSGM

PB2 FTVNVRGSGMRILVR

PB2 ATKRLTVLGKDAGTL

PB2 TVLGKDAGTLTEDPD

PB2 LRGFLILGKENKRYG

PB2 ILGKENKRYGPALSI

PB2 NKRYGPALSINELSN

NS1 MDPNTVSSFQVDCFL

NS1 VDCFLWHVRKRVADQ

NS1 WHVRKRVADQELGDA

NS1 RVADQELGDAPFLDR

NS1 ELGDAPFLDRLRRDQ

NS1 PFLDRLRRDQKSLRG

NS1 LRRDQKSLRGRGSTL

NS1 KSLRGRGSTLGLDIE

NS1 RGSTLGLDIETATRA

NS1 GLDIETATRAGKQIV

NS1 TATRAGKQIVERILK

NS1 ERILKEESDEALKMT

NS1 EESDEALKMTMASVP

NS1 ALKMTMASVPASRYL

NS1 MASVPASRYLTDMTL

NS1 ASRYLTDMTLEEMSR

NS1 TDMTLEEMSREWSML

NS1 EEMSREWSMLIPKQK

NS1 EWSMLIPKQKVAGPL

NS1 IPKQKVAGPLCIRMD

NS1 VAGPLCIRMDQAIMD

NS1 CIRMDQAIMDKNIIL

NS1 KANFSVIFDRLETLI

NS1 VIFDRLETLILLRAF

NS1 SPLPSLPGHTAEDVK

NS1 LPGHTAEDVKNAVGV

NS1 AEDVKNAVGVLIGGL

NS1 NAVGVLIGGLEWNDN

NS1 ETLQRFAWRSSNENG

NS1 FAWRSSNENGRPPLT

NS1 SNENGRPPLTPKQKR

NS1 RPPLTPKQKREMAGT

NS1 PKQKREMAGTIRSEV

NS1 EMAGTIRSEV

NS2 MDPNTVSSFQDILLR

NS2 VSSFQDILLRMSKMQ

NS2 DILLRMSKMQLESSS

NS2 MSKMQLESSSEDLNG

NS2 LESSSEDLNGMITQF

NS2 EDLNGMITQFESLKL

NS2 YRDSLGEAVMRMGDL

NS2 GEAVMRMGDLHSLQN

NS2 RMGDLHSLQNRNEKW

NS2 HSLQNRNEKWREQLG

NS2 IRWLIEEVRHKLKVT

NS2 EEVRHKLKVTENSFE

NS2 KLKVTENSFEQITFM

NS2 ENSFEQITFMQALHL

NS2 QITFMQALHLLLEVE

NS2 QALHLLLEVEQEIRT

NS2 LLEVEQEIRTFSFQL

M1 MSLLTEVETYVLSII

M1 VFAGKNTDLEVLMEW

M1 NTDLEVLMEWLKTRP

M1 VLMEWLKTRPILSPL

M1 QNALNGNGDPNNMDK

M1 GNGDPNNMDKAVKLY

M1 NNMDKAVKLYRKLKR

M1 EITFHGAKEISLSYS

M1 GAKEISLSYSAGALA

M1 SLSYSAGALASCMGL

M1 IYNRMGAVTTEVAFG

M1 GAVTTEVAFGLVCAT

M1 EVAFGLVCATCEQIA

M1 DSQHRSHRQMVTTTN

M1 ASQARQMVQAMRTIG

M1 QMVQAMRTIGTHPSS

M1 THPSSSAGLKNDLLE

M1 SAGLKNDLLENLQAY

M2 IRNEWGCRCNGSSDP

M2 GCRCNGSSDPLTIAA

M2 GSSDPLTIAANIIGI

M2 LTIAANIIGILHLTL

M2 NIIGILHLTLWILDR

M2 LHLTLWILDRLFFKC

M2 WILDRLFFKCIYRRF

M2 IYRRFKYGLKGGPST

M2 KYGLKGGPSTEGVPK

M2 GGPSTEGVPKSMREE

M2 EGVPKSMREEYRKEQ

M2 SMREEYRKEQQSAVD

M2 YRKEQQSAVDADDGH

M2 QSAVDADDGHFVSIE

***HA MKAILVVLLYTFATA***

***HA VVLLYTFATANADTL***

***HA TFATANADTLCIGYH***

***HA NADTLCIGYHANNST***

HA VTVTHSVNLLEDKHN

***HA SVNLLEDKHNGKLCK***

***HA EDKHNGKLCKLRGVA***

***HA GKLCKLRGVAPLHLG***

***HA LRGVAPLHLGKCNIA***

***HA PLHLGKCNIAGWILG***

***HA KCNIAGWILGNPECE***

***HA GWILGNPECESLSTA***

***HA NPECESLSTASSWSY***

***HA SLSTASSWSYIVETP***

***HA SSWSYIVETPSSDNG***

***HA IVETPSSDNGTCYPG***

***HA SSDNGTCYPGDFIDY***

***HA TCYPGDFIDYEELRE***

***HA DFIDYEELREQLSSV***

***HA SSFERFEIFPKTSSW***

***HA FEIFPKTSSWPNHDS***

***HA KTSSWPNHDSNKGVT***

***HA PNHDSNKGVTAACPH***

***HA NKGVTAACPHAGAKS***

***HA AACPHAGAKSFYKNL***

***HA AGAKSFYKNLIWLVK***

***HA FYKNLIWLVKKGNSY***

***HA IWLVKKGNSYPKLSK***

***HA KGNSYPKLSKSYIND***

***HA PKLSKSYINDKGKEV***

***HA SYINDKGKEVLVLWG***

***HA KGKEVLVLWGIHHPS***

***HA LVLWGIHHPSTSADQ***

***HA IHHPSTSADQQSLYQ***

***HA TSADQQSLYQNADTY***

***HA QSLYQNADTYVFVGS***

***HA NADTYVFVGSSRYSK***

***HA VFVGSSRYSKKFKPE***

***HA SRYSKKFKPEIAIRP***

***HA KFKPEIAIRPKVRDQ***

***HA IAIRPKVRDQEGRMN***

HA KVRDQEGRMNYYWTL

HA EGRMNYYWTLVEPGD

***HA YYWTLVEPGDKITFE***

***HA VEPGDKITFEATGNL***

***HA KITFEATGNLVVPRY***

***HA ATGNLVVPRYAFAME***

***HA VVPRYAFAMERNAGS***

***HA AFAMERNAGSGIIIS***

***HA RNAGSGIIISDTPVH***

***HA GIIISDTPVHDCNTT***

***HA DTPVHDCNTTCQTPK***

***HA DCNTTCQTPKGAINT***

***HA CQTPKGAINTSLPFQ***

HA GAINTSLPFQNIHPI

***HA SLPFQNIHPITIGKC***

***HA NIHPITIGKCPKYVK***

***HA TIGKCPKYVKSTKLR***

***HA PKYVKSTKLRLATGL***

***HA STKLRLATGLRNIPS***

HA LATGLRNIPSIQSRG

HA QNEQGSGYAADLKST

***HA SGYAADLKSTQNAID***

***HA DLKSTQNAIDEITNK***

***HA QNAIDEITNKVNSVI***

***HA EITNKVNSVIEKMNT***

***HA QFTAVGKEFNHLEKR***

***HA GKEFNHLEKRIENLN***

***HA HLEKRIENLNKKVDD***

HA IENLNKKVDDGFLDI

HA ELLVLLENERTLDYH

HA LENERTLDYHDSNVK

HA TLDYHDSNVKNLYEK

HA DSNVKNLYEKVRSQL

HA NLYEKVRSQLKNNAK

HA VRSQLKNNAKEIGNG

HA EIGNGCFEFYHKCDN

***HA CFEFYHKCDNTCMES***

***HA HKCDNTCMESVKNGT***

***HA TCMESVKNGTYDYPK***

HA VKNGTYDYPKYSEEA

HA YDYPKYSEEAKLNRE

***HA YSEEAKLNREEIDGV***

***HA KLNREEIDGVKLEST***

***HA EIDGVKLESTRIYQI***

***HA KLESTRIYQILAIYS***

***HA RIYQILAIYSTVASS***

HA LAIYSTVASSLVLVV

HA TVASSLVLVVSLGAI

HA LVLVVSLGAISFWMC

***NA MNPNQKIITIGSVCM***

***NA KIITIGSVCMTIGMA***

***NA GSVCMTIGMANLILQ***

***NA TIGMANLILQIGNII***

***NA NLILQIGNIISIWIS***

***NA IGNIISIWISHSIQL***

***NA SIWISHSIQLGNQNQ***

***NA HSIQLGNQNQIETCN***

***NA GNQNQIETCNQSVIT***

***NA IETCNQSVITYENNT***

***NA QSVITYENNTWVNQT***

***NA YENNTWVNQTYVNIS***

***NA WVNQTYVNISNTNFA***

***NA YVNISNTNFAAGQSV***

***NA NTNFAAGQSVVSVKL***

***NA AGQSVVSVKLAGNSS***

***NA VSVKLAGNSSLCPVS***

***NA AGNSSLCPVSGWAIY***

***NA LCPVSGWAIYSKDNS***

NA GWAIYSKDNSVRIGS

NA SKDNSVRIGSKGDVF

NA VRIGSKGDVFVIREP

***NA VIREPFISCSPLECR***

***NA FISCSPLECRTFFLT***

***NA PLECRTFFLTQGALL***

NA QGALLNDKHSNGTIK

NA NDKHSNGTIKDRSPY

***NA NGTIKDRSPYRTLMS***

***NA DRSPYRTLMSCPIGE***

***NA RTLMSCPIGEVPSPY***

NA CPIGEVPSPYNSRFE

NA VPSPYNSRFESVAWS

NA NSRFESVAWSASACH

***NA SVAWSASACHDGINW***

***NA ASACHDGINWLTIGI***

***NA DGINWLTIGISGPDN***

NA GAVAVLKYNGIITDT

NA LKYNGIITDTIKSWR

***NA IITDTIKSWRNNILR***

***NA IKSWRNNILRTQESE***

***NA NNILRTQESECACVN***

NA TQESECACVNGSCFT

NA CACVNGSCFTVMTDG

***NA GSCFTVMTDGPSNGQ***

***NA VMTDGPSNGQASYKI***

***NA PSNGQASYKIFRIEK***

***NA ASYKIFRIEKGKIVK***

***NA FRIEKGKIVKSVEMN***

***NA GKIVKSVEMNAPNYH***

***NA SVEMNAPNYHYEECS***

***NA APNYHYEECSCYPDS***

***NA YEECSCYPDSSEITC***

***NA CYPDSSEITCVCRDN***

***NA SEITCVCRDNWHGSN***

NA RPWVSFNQNLEYQIG

NA FNQNLEYQIGYICSG

NA EYQIGYICSGIFGDN

NA YICSGIFGDNPRPND

***NA IFGDNPRPNDKTGSC***

***NA PRPNDKTGSCGPVSS***

***NA KTGSCGPVSSNGANG***

***NA GPVSSNGANGVKGFS***

***NA NGANGVKGFSFKYGN***

NA VKGFSFKYGNGVWIG

***NA FKYGNGVWIGRTKSI***

***NA GVWIGRTKSISSRNG***

***NA RTKSISSRNGFEMIW***

***NA SSRNGFEMIWDPNGW***

***NA FEMIWDPNGWTGTDN***

***NA DPNGWTGTDNNFSIK***

***NA TGTDNNFSIKQDIVG***

***NA NFSIKQDIVGINEWS***

***NA QDIVGINEWSGYSGS***

***NA INEWSGYSGSFVQHP***

***NA DCIRPCFWVELIRGR***

***NA CFWVELIRGRPKENT***

***NA LIRGRPKENTIWTSG***

***NA PKENTIWTSGSSISF***

***NA SSISFCGVNSDTVGW***

***NA CGVNSDTVGWSWPDG***

***NA DTVGWSWPDGAELPF***

***NP TKRSYEQMETGGERQ***

***NP EQMETGGERQDATEI***

***NP GGERQDATEIRASVG***

NP DATEIRASVGRMIGG

NP IQMCTELKLSDYDGR

NP ELKLSDYDGRLIQNS

NP DYDGRLIQNSITIER

NP LIQNSITIERMVLSA

NP ITIERMVLSAFDERR

NP KTGGPIYRRVDGKWM

NP IYRRVDGKWMRELIL

NP DGKWMRELILYDKEE

NP RELILYDKEEIRRVW

NP YDKEEIRRVWRQANN

NP IRRVWRQANNGEDAT

NP RQANNGEDATAGLTH

NP GEDATAGLTHIMIWH

NP IMIWHSNLNDATYQR

NP SGAAGAAVKGVGTIA

***NP AAVKGVGTIAMELIR***

***NP VGTIAMELIRMIKRG***

NP MELIRMIKRGINDRN

NP INDRNFWRGENGRRT

NP FWRGENGRRTRVAYE

NP NGRRTRVAYERMCNI

NP RVAYERMCNILKGKF

NP LKGKFQTAAQRAMMD

NP QTAAQRAMMDQVRES

NP RAMMDQVRESRNPGN

NP RNPGNAEIEDLIFLA

NP AEIEDLIFLARSALI

NP LIFLARSALILRGSV

NP AHKSCLPACVYGLAV

***NP LPACVYGLAVASGHD***

***NP YGLAVASGHDFEREG***

NP ASGHDFEREGYSLVG

NP FEREGYSLVGIDPFK

NP YSLVGIDPFKLLQNS

***NP IDPFKLLQNSQVVSL***

***NP LLQNSQVVSLMRPNE***

***NP QVVSLMRPNENPAHK***

NP MRPNENPAHKSQLVW

NP MACHSAAFEDLRVSS

***NP AAFEDLRVSSFIRGK***

***NP LRVSSFIRGKKVIPR***

***NP FIRGKKVIPRGKLST***

NP KVIPRGKLSTRGVQI

NP RGVQIASNENVETMD

***NP ASNENVETMDSNTLE***

***NP VETMDSNTLELRSRY***

NP SNTLELRSRYWAIRT

NP WAIRTRSGGNTNQQK

NP RSGGNTNQQKASAGQ

NP TNQQKASAGQISVQP

NP ASAGQISVQPTFSVQ

NP ISVQPTFSVQRNLPF

***NP TFSVQRNLPFERATV***

***NP RNLPFERATVMAAFS***

***NP ERATVMAAFSGNNEG***

***NP MAAFSGNNEGRTSDM***

NP GNNEGRTSDMRTEVI

NP RTSDMRTEVIRMMES

NP RTEVIRMMESAKPED

NP RMMESAKPEDLSFQG

NP AKPEDLSFQGRGVFE

NP LSFQGRGVFELSDEK

NP RGVFELSDEKATNPI

NP LSDEKATNPIVPSFD

NP SYFFGDNAEEYDS

***PA LAEKAMKEYGEDPKI***

***PA MKEYGEDPKIETNKF***

***PA EDPKIETNKFAAICT***

PA HLEVCFMYSDFHFID

***PA FMYSDFHFIDERGES***

***PA FHFIDERGESIIVES***

***PA ERGESIIVESGDPNA***

PA IIVESGDPNALLKHR

PA LLKHRFEIIEGRDRI

PA FEIIEGRDRIMAWTV

PA GRDRIMAWTVVNSIC

PA MAWTVVNSICNTTGV

PA VNSICNTTGVEKPKF

PA NTTGVEKPKFLPDLY

PA FTIRQEMASRSLWDS

PA EMASRSLWDSFRQSE

PA SLWDSFRQSERGEET

PA FRQSERGEETIEEKF

PA RGEETIEEKFEITGT

PA IEEKFEITGTMRKLA

***PA MRKLADQSLPPNFPS***

***PA DQSLPPNFPSLENFR***

***PA PNFPSLENFRAYVDG***

***PA AYVDGFEPNGCIEGK***

***PA FEPNGCIEGKLSQMS***

***PA CIEGKLSQMSKEVNA***

PA LSQMSKEVNAKIEPF

PA KEVNAKIEPFLRTTP

PA KIEPFLRTTPRPLRL

***PA LRTTPRPLRLPDGPL***

***PA RPLRLPDGPLCHQRS***

***PA PDGPLCHQRSKFLLM***

***PA CHQRSKFLLMDALKL***

PA GIPLYDAIKCMKTFF

PA DAIKCMKTFFGWKEP

PA MKTFFGWKEPNIVKP

PA GWKEPNIVKPHEKGI

PA NIVKPHEKGINPNYL

***PA HEKGINPNYLMAWKQ***

***PA NPNYLMAWKQVLAEL***

***PA MAWKQVLAELQDIEN***

PA QDIENEEKIPRTKNM

PA EEKIPRTKNMKRTSQ

PA RTKNMKRTSQLKWAL

PA KRTSQLKWALGENMA

***PA DVGDLKQYDSDEPEP***

***PA KQYDSDEPEPRSLAS***

***PA DEPEPRSLASWVQNE***

PA RSLASWVQNEFNKAC

PA WVQNEFNKACELTDS

PA CVLEIGDMLLRTAIG

PA GDMLLRTAIGQVSRP

PA RTAIGQVSRPMFLYV

PA SETWPIGESPRGVEE

PA IGESPRGVEEGSIGK

PA RGVEEGSIGKVCRTL

PA WFNSFLTHALK

PB1 MDVNPTLLFLKIPAQ

PB1 TLLFLKIPAQNAIST

PB1 KIPAQNAISTTFPYT

PB1 DTVNRTHQYSEKGKW

PB1 THQYSEKGKWTTNTE

PB1 EKGKWTTNTETGAPQ

PB1 EESHPGIFENSCLET

PB1 GIFENSCLETMEVVQ

PB1 SCLETMEVVQQTRVD

***PB1 FLKDVMESMNKEEIE***

***PB1 MESMNKEEIEITTHF***

PB1 KEEIEITTHFQRKRR

PB1 QRKRRVRDNMTKKMV

PB1 VRDNMTKKMVTQRTI

PB1 TKKMVTQRTIGKKKQ

PB1 TQRTIGKKKQRLNKR

PB1 GKKKQRLNKRGYLIR

PB1 RLNKRGYLIRALTLN

PB1 GYLIRALTLNTMTKD

PB1 RMFLAMITYITRNQP

PB1 MITYITRNQPEWFRN

PB1 TRNQPEWFRNILSMA

PB1 EWFRNILSMAPIMFS

PB1 ILSMAPIMFSNKMAR

***PB1 LGKGYMFESKRMKIR***

***PB1 MFESKRMKIRTQIPA***

***PB1 RMKIRTQIPAEMLAS***

PB1 EMLASIDLKYFNEST

PB1 IDLKYFNESTKKKIE

PB1 FNESTKKKIEKIRPL

PB1 STVLGVSILNLGQKK

***PB1 VSILNLGQKKYTKTI***

***PB1 LGQKKYTKTIYWWDG***

PB1 YTKTIYWWDGLQSSD

PB1 AGVDRFYRTCKLVGI

PB1 FYRTCKLVGINMSKK

PB1 KLVGINMSKKKSYIN

PB1 NMSKKKSYINKTGTF

PB1 KSYINKTGTFEFTSF

PB1 KTGTFEFTSFFYRYG

PB1 TQIQTRRSFELKKLW

PB1 RRSFELKKLWDQTQS

***PB1 LKKLWDQTQSKVGLL***

***PB1 DQTQSKVGLLVSDGG***

PB1 KVGLLVSDGGPNLYN

PB1 IPEVCLKWELMDDDY

***PB1 LKWELMDDDYRGRLC***

***PB1 MDDDYRGRLCNPLNP***

PB1 RGRLCNPLNPFVSHK

PB1 NPLNPFVSHKEIDSV

PB1 FVSHKEIDSVNNAVV

PB1 EIDSVNNAVVMPAHG

PB1 NNAVVMPAHGPAKSM

PB1 MPAHGPAKSMEYDAV

PB1 PAKSMEYDAVATTHS

PB1 GILEDEQMYQKCCNL

PB1 EQMYQKCCNLFEKFF

PB1 KCCNLFEKFFPSSSY

PB1 EAMVSRARIDARVDF

PB1 RARIDARVDFESGRI

PB1 ARVDFESGRIKKEEF

PB1 ESGRIKKEEFSEIMK

PB1 KKEEFSEIMKICSTI

PB1 SEIMKICSTIEELRR

PB2 MERIKELRDLMSQSR

PB2 ELRDLMSQSRTREIL

PB2 KNPALRMKWMMAMRY

PB2 RMKWMMAMRYPITAD

***PB2 MAMRYPITADKRIMD***

***PB2 PITADKRIMDMIPER***

***PB2 KRIMDMIPERNEQGQ***

PB2 NEQGQTLWSKTNDAG

PB2 TLWSKTNDAGSDRVM

***PB2 TNDAGSDRVMVSPLA***

PB2 VSPLAVTWWNRNGPT

***PB2 VTWWNRNGPTTSTVH***

***PB2 RNGPTTSTVHYPKVY***

PB2 TSTVHYPKVYKTYFE

PB2 YPKVYKTYFEKVERL

PB2 RNQVKIRRRVDTNPG

PB2 IRRRVDTNPGHADLS

PB2 DTNPGHADLSAKEAQ

PB2 EVGARILTSESQLAI

PB2 ILTSESQLAITKEKK

PB2 SQLAITKEKKEELQD

PB2 TKEKKEELQDCKIAP

PB2 EELQDCKIAPLMVAY

PB2 CKIAPLMVAYMLERE

PB2 LVRKTRFLPVAGGTG

PB2 RFLPVAGGTGSVYIE

PB2 AGGTGSVYIEVLHLT

PB2 LEMCHSTQIGGVRMV

PB2 STQIGGVRMVDILRQ

PB2 GVRMVDILRQNPTEE

PB2 NPTEEQAVDICKAAI

PB2 QAVDICKAAIGLRIS

PB2 CKAAIGLRISSSFSF

PB2 GGFTFKRTSGSSVKK

PB2 KRTSGSSVKKEEEVL

PB2 SSVKKEEEVLTGNLQ

***PB2 DAKVLFQNWGIESID***

***PB2 FQNWGIESIDNVMGM***

PB2 IESIDNVMGMIGILP

PB2 IGILPDMTPSTEMSL

PB2 DMTPSTEMSLRGIRV

PB2 TEMSLRGIRVSKMGV

PB2 RGIRVSKMGVDEYSS

PB2 TERVVVSIDRFLRVR

PB2 LVNTYQWIIRNWEIV

PB2 QWIIRNWEIVKIQWS

***PB2 NWEIVKIQWSQDPTM***

PB2 KIQWSQDPTMLYNKM

PB2 QDPTMLYNKMEFEPF

***PB2 EFEPFQSLVPKATRS***

***PB2 QSLVPKATRSRYSGF***

***PB2 KATRSRYSGFVRTLF***

PB2 RYSGFVRTLFQQMRD

PB2 QQMRDVLGTFDTVQI

PB2 VLGTFDTVQIIKLLP

PB2 DTVQIIKLLPFAAAP

PB2 IKLLPFAAAPPEQSR

PB2 FAAAPPEQSRMQFSS

PB2 PEQSRMQFSSLTVNV

PB2 MQFSSLTVNVRGSGL

PB2 LTVNVRGSGLRILVR

PB2 RGSGLRILVRGNSPV

PB2 ATKRLTVLGKDAGAL

PB2 TVLGKDAGALTEDPD

***PB2 DAGALTEDPDEGTSG***

PB2 TEDPDEGTSGVESAV

PB2 EGTSGVESAVLRGFL

PB2 LRGFLILGKEDKRYG

PB2 ILGKEDKRYGPALSI

PB2 DKRYGPALSINELSN

***NS1 MDSNTMSSFQVDCFL***

NS1 MSSFQVDCFLWHIRK

***NS1 VDCFLWHIRKRFADN***

***NS1 WHIRKRFADNGLGDA***

***NS1 RFADNGLGDAPFLDR***

***NS1 GLGDAPFLDRLRRDQ***

NS1 LRRDQKSLKGRGNTL

NS1 KSLKGRGNTLGLDIE

***NS1 RGNTLGLDIETATLV***

***NS1 GLDIETATLVGKQIV***

***NS1 TATLVGKQIVEWILK***

***NS1 GKQIVEWILKEESSE***

***NS1 EWILKEESSETLRMT***

***NS1 EESSETLRMTIASVP***

***NS1 TLRMTIASVPTSRYL***

***NS1 IASVPTSRYLSDMTL***

***NS1 TSRYLSDMTLEEMSR***

NS1 SDMTLEEMSRDWFML

NS1 EEMSRDWFMLMPRQK

***NS1 DWFMLMPRQKIIGPL***

***NS1 MPRQKIIGPLCVRLD***

***NS1 IIGPLCVRLDQAIME***

NS1 CVRLDQAIMEKNIVL

NS1 QAIMEKNIVLKANFS

NS1 KNIVLKANFSVIFNR

NS1 KANFSVIFNRLETLI

NS1 VIFNRLETLILLRAF

***NS1 SPLPSLPGHTYEDVK***

***NS1 LPGHTYEDVKNAVGV***

***NS1 YEDVKNAVGVLIGGL***

***NS1 NAVGVLIGGLEWNGN***

***NS1 LIGGLEWNGNTVRVS***

***NS1 EWNGNTVRVSENIQR***

***NS1 TVRVSENIQRFAWRN***

***NS1 ENIQRFAWRNCDENG***

***NS1 FAWRNCDENGRPSLP***

***NS1 CDENGRPSLPPEQK***

***NS2 MDSNTMSSFQDILMR***

NS2 MSSFQDILMRMSKMQ

***NS2 MSKMQLGSSSEDLNG***

***NS2 LGSSSEDLNGMVTRF***

***NS2 EDLNGMVTRFESLKI***

***NS2 MVTRFESLKIYRDSL***

***NS2 ESLKIYRDSLGETVM***

***NS2 YRDSLGETVMRMGDL***

***NS2 GETVMRMGDLHYLQS***

***NS2 RMGDLHYLQSRNEKW***

***NS2 HYLQSRNEKWREQLG***

NS2 QKFEEIRWLIEEMRH

***NS2 IRWLIEEMRHRLKAT***

***NS2 EEMRHRLKATENSFE***

NS2 RLKATENSFEQITFM

NS2 QITFMQALQLLLEVE

***NS2 QALQLLLEVEQEIRA***

NS2 LLEVEQEIRAFSFQL

NS2 QEIRAFSFQLI

M1 LKAEIAQRLESVFAG

M1 AQRLESVFAGKNTDL

M1 SVFAGKNTDLEALME

M1 PNNMDRAVKLYKKLK

M1 RAVKLYKKLKREITF

M1 YKKLKREITFHGAKE

M1 REITFHGAKEVSLSY

***M1 HGAKEVSLSYSTGAL***

***M1 VSLSYSTGALASCMG***

***M1 STGALASCMGLIYNR***

***M1 ASCMGLIYNRMGTVT***

***M1 LIYNRMGTVTTEAAF***

***M1 MGTVTTEAAFGLVCA***

M1 TEAAFGLVCATCEQI

M1 ADSQHRSHRQMATTT

M1 RSHRQMATTTNPLIR

M1 MATTTNPLIRHENRM

***M1 SSEQAAEAMEVANQT***

***M1 AEAMEVANQTRQMVH***

***M1 VANQTRQMVHAMRTI***

M1 RQMVHAMRTIGTHPS

M1 GTHPSSSAGLKDDLL

M1 SSAGLKDDLLENLQA

M1 KDDLLENLQAYQKRM

***M2 TEVETPTRSEWECRC***

***M2 PTRSEWECRCSDSSD***

***M2 WECRCSDSSDPLVIA***

***M2 SDSSDPLVIAANIIG***

***M2 PLVIAANIIGILHLI***

***M2 ANIIGILHLILWITD***

***M2 ILHLILWITDRLFFK***

***M2 LWITDRLFFKCIYRR***

***M2 CIYRRFKYGLKRGPS***

M2 FKYGLKRGPSTEGVP

***M2 TEGVPESMREEYQQE***

***M2 ESMREEYQQEQQSAV***

***M2 EYQQEQQSAVDVDDG***

***M2 QQSAVDVDDGHFVNI***

***M2 DVDDGHFVNIELE***

***M2 AVDVDDGHFVNIELE***

HA LVLLCTFTATYADTI

HA TFTATYADTICIGYH

HA YADTICIGYHANNST

HA GKLCLLKGIAPLQLG

HA LKGIAPLQLGNCSVA

HA PLQLGNCSVAGWILG

HA NCSVAGWILGNPECE

HA GWILGNPECELLISK

HA NPECELLISKESWSY

HA SSFERFEIFPKESSW

HA FEIFPKESSWPNHTV

HA KESSWPNHTVTGVSA

HA PNHTVTGVSASCSHN

HA YRNLLWLTGKNGLYP

HA WLTGKNGLYPNLSKS

HA EKEVLVLWGVHHPPN

HA ALYHTENAYVSVVSS

HA FTPEIAKRPKVRDQE

HA AKRPKVRDQEGRINY

HA VRDQEGRINYYWTLL

HA GRINYYWTLLEPGDT

HA YWTLLEPGDTIIFEA

HA EPGDTIIFEANGNLI

HA CDAKCQTPQGAINSS

HA QTPQGAINSSLPFQN

HA AINSSLPFQNVHPVT

HA LPFQNVHPVTIGECP

HA VHPVTIGECPKYVRS

HA IGECPKYVRSAKLRM

HA KYVRSAKLRMVTGLR

HA AKLRMVTGLRNIPSI

HA VTGLRNIPSIQSRGL

HA NEQGSGYAADQKSTQ

HA GYAADQKSTQNAING

HA QKSTQNAINGITNKV

HA NAINGITNKVNSVIE

HA KMNTQFTAVGKEFNK

HA FTAVGKEFNKLERRM

HA KEFNKLERRMENLNK

HA LERRMENLNKKVDDG

HA LLVLLENERTLDFHD

HA ENERTLDFHDSNVKN

HA LDFHDSNVKNLYEKV

HA SNVKNLYEKVKSQLK

HA LYEKVKSQLKNNAKE

HA KSQLKNNAKEIGNGC

HA KNGTYDYPKYSEESK

HA DYPKYSEESKLNREK

HA SEESKLNREKIDGVK

HA LNREKIDGVKLESMG

HA IDGVKLESMGVYQIL

HA LESMGVYQILAIYST

HA VYQILAIYSTVASSL

HA AIYSTVASSLVLLVS

HA VASSLVLLVSLGAIS

HA VLLVSLGAISFWMCS

HA VLLCTFTATYADTICIG

HA TATYADTICIGYHANNS

HA TICIGYHANNSTDTVDT

HA HNGKLCLLKGIAPLQLG

HA LLKGIAPLQLGNCSVAG

HA PLQLGNCSVAGWILGNP

HA CSVAGWILGNPECELLI

HA ILGNPECELLISKESWS

HA SSFERFEIFPKESSWPN

HA EIFPKESSWPNHTVTGV

HA SSWPNHTVTGVSASCSH

HA RNLLWLTGKNGLYPNLS

HA EKEVLVLWGVHHPPNIG

HA TPEIAKRPKVRDQEGRI

HA RPKVRDQEGRINYYWTL

HA DQEGRINYYWTLLEPGD

HA NYYWTLLEPGDTIIFEA

HA LEPGDTIIFEANGNLIA

HA DAKCQTPQGAINSSLPF

HA PQGAINSSLPFQNVHPV

HA SSLPFQNVHPVTIGECP

HA NVHPVTIGECPKYVRSA

HA IGECPKYVRSAKLRMVT

HA YVRSAKLRMVTGLRNIP

HA LRMVTGLRNIPSIQSRG

HA HQNEQGSGYAADQKSTQ

HA SGYAADQKSTQNAINGI

HA DQKSTQNAINGITNKVN

HA NAINGITNKVNSVIEKM

HA TQFTAVGKEFNKLERRM

HA GKEFNKLERRMENLNKK

HA ELLVLLENERTLDFHDS

HA ENERTLDFHDSNVKNLY

HA DFHDSNVKNLYEKVKSQ

HA VKNLYEKVKSQLKNNAK

HA KVKSQLKNNAKEIGNG

HA GTYDYPKYSEESKLNRE

HA KYSEESKLNREKIDGVK

HA KLNREKIDGVKLESMGV

HA IDGVKLESMGVYQILAI

HA ESMGVYQILAIYSTVAS

HA YQILAIYSTVASSLVLL

HA YSTVASSLVLLVSLGAI

HA SLVLLVSLGAISFWMCS

NA MNPNQKIITIGSISI

NA KIITIGSISIAIGII

NA GSISIAIGIISLMLQ

NA AIGIISLMLQIGNII

NA SLMLQIGNIISIWAS

NA IGNIISIWASHSIQT

NA QRIITYENSTWVNHT

NA YENSTWVNHTYVNIN

NA WVNHTYVNINNTNVV

NA TSVTLAGNSSLCSIS

NA AGNSSLCSISGWAIY

NA LCSISGWAIYTKDNS

NA GWAIYTKDNSIRIGS

NA TKDNSIRIGSKGDVF

NA IRIGSKGDVFVIREP

NA VIREPFISCSHLECR

NA FISCSHLECRTFFLT

NA HLECRTFFLTQGALL

NA QGALLNDKHSNGTVK

NA NDKHSNGTVKDRSPY

NA NGTVKDRSPYRALMS

NA DRSPYRALMSCPLGE

NA RALMSCPLGEAPSPY

NA CPLGEAPSPYNSKFE

NA APSPYNSKFESVAWS

NA NSKFESVAWSASACH

NA SVAWSASACHDGMGW

NA ASACHDGMGWLTIGI

NA DGMGWLTIGISGPDN

NA ASYKIFKIEKGKVTK

NA FKIEKGKVTKSIELN

NA GKVTKSIELNAPNFH

NA SIELNAPNFHYEECS

NA APNFHYEECSCYPDT

NA RPWVSFNQNLDYQIG

NA FNQNLDYQIGYICSG

NA DYQIGYICSGVFGDN

NA GVWIGRTKSNRLRKG

NA RTKSNRLRKGFEMIW

NA RLRKGFEMIWDPNGW

NA DFSVKQDVVAITDWS

NA QDVVAITDWSGYSGS

NA ITDWSGYSGSFVQHP

NA DCIRPCFWVELVRGL

NA CFWVELVRGLPRENT

NA LVRGLPRENTTIWTS

NA PRENTTIWTSGSSIS

NA GSSISFCGVNSDTAN

NA FCGVNSDTANWSWPD

NA SDTANWSWPDGAELP

NA MNPNQKIITIGSISIAI

NA IITIGSISIAIGIISLM

NA ISIAIGIISLMLQIGNI

NA IISLMLQIGNIISIWAS

NA LQIGNIISIWASHSIQT

NA ITYENSTWVNHTYVNIN

NA TWVNHTYVNINNTNVVA

NA TLAGNSSLCSISGWAIY

NA SLCSISGWAIYTKDNSI

NA GWAIYTKDNSIRIGSKG

NA KDNSIRIGSKGDVFVIR

NA DVFVIREPFISCSHLE

NA REPFISCSHLECRTFFL

NA CSHLECRTFFLTQGALL

NA TQGALLNDKHSNGTVKD

NA NDKHSNGTVKDRSPYRA

NA GTVKDRSPYRALMSCPL

NA SPYRALMSCPLGEAPSP

NA MSCPLGEAPSPYNSKFE

NA EAPSPYNSKFESVAWSA

NA NSKFESVAWSASACHDG

NA VAWSASACHDGMGWLTI

NA ACHDGMGWLTIGISGPD

NA GWLTIGISGPDNGAVAV

NA IFKIEKGKVTKSIELNA

NA GKVTKSIELNAPNFHYE

NA IELNAPNFHYEECSCYP

NA HGSNRPWVSFNQNLDYQ

NA WVSFNQNLDYQIGYICS

NA NLDYQIGYICSGVFGDN

NA VWIGRTKSNRLRKGFEM

NA KSNRLRKGFEMIWDPNG

NA FSVKQDVVAITDWSGYS

NA VVAITDWSGYSGSFVQH

NA DCIRPCFWVELVRGLPR

NA FWVELVRGLPRENTTIW

NA RGLPRENTTIWTSGSSI

NA NTTIWTSGSSISFCGVN

NA SGSSISFCGVNSDTANW

NA FCGVNSDTANWSWPDGA

NP TKRSYEQMETDGERQ

NP EQMETDGERQNATEI

NP DGERQNATEIRASVG

NP DYEGRLIQNSLTIER

NP LIQNSLTIERMVLSA

NP LTIERMVLSAFDERR

NP YDKEEIRRIWRQANN

NP IRRIWRQANNGDDAT

NP RQANNGDDATAGLTH

NP SGAAGAAVKGVGTMV

NP INDRNFWRGENGRKT

NP FWRGENGRKTRIAYE

NP NGRKTRIAYERMCNI

NP RIAYERMCNILKGKF

NP LKGKFQTAAQKAMMD

NP QTAAQKAMMDQVRES

NP KAMMDQVRESRNPGN

NP LTFLARSALILRGSV

NP AHKSCLPACVYGPAV

NP LPACVYGPAVASGYD

NP QVYSLIRPNENPAHK

NP IRPNENPAHKSQLVW

NP SSTLELRSRYWAIRT

NP WAIRTRSGGNTNQQR

NP RSGGNTNQQRASAGQ

NP SYFFGDNAEEYDN

NP MASQGTKRSYEQMETDG

NP KRSYEQMETDGERQNAT

NP METDGERQNATEIRASV

NP RLIQNSLTIERMVLSAF

NP LTIERMVLSAFDERRNK

NP EIRRIWRQANNGDDATA

NP RGINDRNFWRGENGRKT

NP NFWRGENGRKTRIAYER

NP NGRKTRIAYERMCNILK

NP IAYERMCNILKGKFQTA

NP CNILKGKFQTAAQKAMM

NP KFQTAAQKAMMDQVRES

NP AQKAMMDQVRESRNPGN

NP VAHKSCLPACVYGPAVA

NP LPACVYGPAVASGYDFE

NP VYSLIRPNENPAHKSQL

NP RYWAIRTRSGGNTNQQR

NP TRSGGNTNQQRASAGQI

PA MKEYGEDLKIETNKF

PA EDLKIETNKFAAICT

PA HLEVCFMYSDFHFIN

PA FMYSDFHFINEQGES

PA LLKHRFEIIEGRDRT

PA FEIIEGRDRTMAWTV

PA GRDRTMAWTVVNSIC

PA MAWTVVNSICNTTGA

PA VNSICNTTGAEKPKF

PA NTTGAEKPKFLPDLY

PA FTIRQEMASRGLWDS

PA EMASRGLWDSFRQSE

PA GLWDSFRQSERGEET

PA FRQSERGEETIEERF

PA RGEETIEERFEITGT

PA AYVDGFEPNGYIEGK

PA FEPNGYIEGKLSQMS

PA YIEGKLSQMSKEVNA

PA LSQMSKEVNARIEPF

PA KEVNARIEPFLKTTP

PA GIPLYDAIKCMRTFF

PA DAIKCMRTFFGWKEP

PA HEKGINPNYLLSWKQ

PA KKTSQLKWALGENMA

PA WIQNEFNKACELTDS

PA RSAIGQVSRPMFLYV

PA SETWPIGESPKGVEE

PA YGEDLKIETNKFAAICT

PA LEVCFMYSDFHFINEQG

PA YSDFHFINEQGESIIVE

PA LKHRFEIIEGRDRTMAW

PA IIEGRDRTMAWTVVNSI

PA RTMAWTVVNSICNTTGA

PA VVNSICNTTGAEKPKFL

PA NTTGAEKPKFLPDLYDY

PA RLFTIRQEMASRGLWDS

PA RQEMASRGLWDSFRQSE

PA RGLWDSFRQSERGEETI

PA FRQSERGEETIEERFEI

PA YVDGFEPNGYIEGKLSQ

PA PNGYIEGKLSQMSKEVN

PA GKLSQMSKEVNARIEPF

PA SKEVNARIEPFLKTTPR

PA EGEGIPLYDAIKCMRTF

PA LYDAIKCMRTFFGWKEP

PA VVKPHEKGINPNYLLSW

PA TKNMKKTSQLKWALGEN

PA SAIGQVSRPMFLYVRTN

PB1 MDVNPTLLFLKVPAQ

PB1 TLLFLKVPAQNAIST

PB1 KVPAQNAISTTFPYT

PB1 EESHPGIFENSCIET

PB1 GIFENSCIETMEVVQ

PB1 SCIETMEVVQQTRVD

PB1 SYLIRALTLNTMTKD

PB1 LGKGYMFESKSMKLR

PB1 MFESKSMKLRTQIPA

PB1 STVLGVSILNLGQKR

PB1 VSILNLGQKRYTKTT

PB1 LGQKRYTKTTYWWDG

PB1 YTKTTYWWDGLQSSD

PB1 AGVDRFYRTCKLLGI

PB1 FYRTCKLLGINMSKK

PB1 KLLGINMSKKKSYIN

PB1 NMSKKKSYINRTGTF

PB1 KSYINRTGTFEFTSF

PB1 RTGTFEFTSFFYRYG

PB1 IPEVCLKWELMDEDY

PB1 LKWELMDEDYQGRLC

PB1 MDEDYQGRLCNPLNP

PB1 QGRLCNPLNPFVSHK

PB1 MPAHGPAKNMEYDAV

PB1 PAKNMEYDAVATTHS

PB1 EQMYQRCCNLFEKFF

PB1 RCCNLFEKFFPSSSY

PB1 EAMVSRARIDARIDF

PB1 RARIDARIDFESGRI

PB1 ARIDFESGRIKKEEF

PB1 MDVNPTLLFLKVPAQNA

PB1 LLFLKVPAQNAISTTFP

PB1 EESHPGIFENSCIETME

PB1 IFENSCIETMEVVQQTR

PB1 IETMEVVQQTRVDKLTQ

PB1 RSYLIRALTLNTMTKDA

PB1 NKMARLGKGYMFESKSM

PB1 GKGYMFESKSMKLRTQI

PB1 ESKSMKLRTQIPAEMLA

PB1 VLGVSILNLGQKRYTKT

PB1 LNLGQKRYTKTTYWWDG

PB1 RYTKTTYWWDGLQSSDD

PB1 IQAGVDRFYRTCKLLGI

PB1 RFYRTCKLLGINMSKKK

PB1 KLLGINMSKKKSYINRT

PB1 MSKKKSYINRTGTFEFT

PB1 YINRTGTFEFTSFFYRY

PB1 RNLHIPEVCLKWELMDE

PB1 EVCLKWELMDEDYQGRL

PB1 ELMDEDYQGRLCNPLNP

PB1 YQGRLCNPLNPFVSHKE

PB1 GPAKNMEYDAVATTHSW

PB1 EQMYQRCCNLFEKFFPS

PB1 EAMVSRARIDARIDFES

PB1 ARIDARIDFESGRIKKE

PB2 MERIKELRNLMSQSR

PB2 ELRNLMSQSRTREIL

PB2 RMKWMMAMKYPITAD

PB2 MAMKYPITADKRITE

PB2 PITADKRITEMIPER

PB2 KRITEMIPERNEQGQ

PB2 RNQVKIRRRVDINPG

PB2 IRRRVDINPGHADLS

PB2 DINPGHADLSAKEAQ

PB2 EVGARILTSESQLTI

PB2 ILTSESQLTITKEKK

PB2 CKISPLMVAYMLERE

PB2 LVRKTRFLPVAGGTS

PB2 RFLPVAGGTSSVYIE

PB2 AGGTSSVYIEVLHLT

PB2 NPTEEQAVDICKAAM

PB2 QAVDICKAAMGLRIS

PB2 CKAAMGLRISSSFSF

PB2 GGFTFKRTSGSSVKR

PB2 KRTSGSSVKREEEVL

PB2 SSVKREEEVLTGNLQ

PB2 QWIIRNWETVKIQWS

PB2 NWETVKIQWSQNPTM

PB2 KIQWSQNPTMLYNKM

PB2 QNPTMLYNKMEFEPF

PB2 EFEPFQSLVPKAIRG

PB2 QSLVPKAIRGQYSGF

PB2 KAIRGQYSGFVRTLF

PB2 QYSGFVRTLFQQMRD

PB2 IKLLPFAAAPPKQSR

PB2 FAAAPPKQSRMQFSS

PB2 RGSGMRILVRGNSPV

PB2 DAGTLTEDPDEGTAG

PB2 TEDPDEGTAGVESAV

PB2 EGTAGVESAVLRGFL

PB2 MERIKELRNLMSQSRTR

PB2 LRNLMSQSRTREILTKT

PB2 KWMMAMKYPITADKRIT

PB2 KYPITADKRITEMIPER

PB2 DKRITEMIPERNEQGQT

PB2 PVHFRNQVKIRRRVDIN

PB2 NQVKIRRRVDINPGHAD

PB2 RRVDINPGHADLSAKEA

PB2 GARILTSESQLTITKEK

PB2 KISPLMVAYMLERELVR

PB2 RELVRKTRFLPVAGGTS

PB2 TRFLPVAGGTSSVYIEV

PB2 AGGTSSVYIEVLHLTQG

PB2 TEEQAVDICKAAMGLRI

PB2 DICKAAMGLRISSSFSF

PB2 MGLRISSSFSFGGFTFK

PB2 GFTFKRTSGSSVKREEE

PB2 TSGSSVKREEEVLTGNL

PB2 NTYQWIIRNWETVKIQW

PB2 IRNWETVKIQWSQNPTM

PB2 VKIQWSQNPTMLYNKME

PB2 SQNPTMLYNKMEFEPFQ

PB2 FEPFQSLVPKAIRGQYS

PB2 LVPKAIRGQYSGFVRTL

PB2 RGQYSGFVRTLFQQMRD

PB2 LLPFAAAPPKQSRMQFS

PB2 GSGMRILVRGNSPVFNY

PB2 AGTLTEDPDEGTAGVES

PB2 DPDEGTAGVESAVLRGF

PB2 AGVESAVLRGFLILGKE

NS1 VSSFQVDCFLWHVRK

NS1 GKQIVERILKEESDE

NS1 QAIMDKNIILKANFS

NS1 KNIILKANFSVIFDR

NS1 LIGGLEWNDNTVRVS

NS1 EWNDNTVRVSETLQR

NS1 TVRVSETLQRFAWRS

NS1 DQAIMDKNIILKANFSV

NS1 KNIILKANFSVIFDRLE

NS1 VLIGGLEWNDNTVRVSE

NS1 EWNDNTVRVSETLQRFA

NS1 VRVSETLQRFAWRSSNE

NS2 MITQFESLKLYRDSL

NS2 ESLKLYRDSLGEAVM

NS2 QKFEEIRWLIEEVRH

NS2 QEIRTFSFQLI

NS2 MITQFESLKLYRDSLGE

NS2 EQLGQKFEEIRWLIEEV

NS2 VEQEIRTFSFQLI

M1 PSGPLKAEIAQRLED

M1 KAEIAQRLEDVFAGK

M1 QRLEDVFAGKNTDLE

M1 AVKLYRKLKREITFH

M1 RKLKREITFHGAKEI

M1 AGALASCMGLIYNRM

M1 SCMGLIYNRMGAVTT

M1 SHRQMVTTTNPLIRH

M1 VTTTNPLIRHENRMV

M1 SEQAAEAMEVASQAR

M1 EAMEVASQARQMVQA

M1 NDLLENLQAYQKRMG

M1 PLKAEIAQRLEDVFAGK

M1 VKLYRKLKREITFHGAK

M1 AGALASCMGLIYNRMGA

M1 RQMVTTTNPLIRHENRM

M1 AGSSEQAAEAMEVASQA

M1 AAEAMEVASQARQMVQA

M1 GLKNDLLENLQAYQKRM

M2 MSLLTEVETPIRNEW

M2 EVETPIRNEWGCRCN

M2 ADDGHFVSIELE

M2 MSLLTEVETPIRNEWG

M2 VDADDGHFVSIELE

HA NLNKKVDDGFLDIWTYN

HA DDGFLDIWTYNAELLVL

HA KKVDDGFLDIWTYNA

HA GFLDIWTYNAELLVL

NA WPDGAELPFTIDK

NA SWPDGAELPFTIDK

NP IRASVGRMIGGIGRFYI

NP RMIGGIGRFYIQMCTEL

NP LIRMIKRGINDRNFWRG

NP DQVRESRNPGNAEIEDL

NP RASVGRMIGGIGRFY

NP RMIGGIGRFYIQMCT

NP AGLTHIMIWHSNLND

NP QVRESRNPGNAEIED

NP ATNPIVPSFDMSNEG

PA RQCFNPMIVELAEKAMK

PA PIEHIASMRRNYFTAEV

PA SMRRNYFTAEVSHCRAT

PA FTAEVSHCRATEYIMKG

PA PHKWEKYCVLEIGDMLL

PA EGSIGKVCRTLLAKSVF

PA RQCFNPMIVELAEKA

PA PMIVELAEKAMKEYG

PA EHIASMRRNYFTAEV

PA MRRNYFTAEVSHCRA

PA FTAEVSHCRATEYIM

PA KWEKYCVLEIGDMLL

PA GSIGKVCRTLLAKSV

PB1 RKMMTNSQDTEISFTIT

PB1 SQDTEISFTITGDNTKW

PB1 KIEKIRPLLIDGTASLS

PB1 PLLIDGTASLSPGMMMG

PB1 FVANFSMELPSFGVSGV

PB1 MELPSFGVSGVNESADM

PB1 GVSGVNESADMSIGVTV

PB1 IGVTVIKNNMINNDLGP

PB1 KNNMINNDLGPATAQMA

PB1 NDLGPATAQMALQLFIK

PB1 TAQMALQLFIKDYRYTY

PB1 LQLFIKDYRYTYRCHRG

PB1 DYRYTYRCHRGDTQIQT

PB1 LVSDGGPNLYNIRNLHI

PB1 PNLYNIRNLHIPEVCLK

PB1 PKRNRSILNTSQRGILE

PB1 ILNTSQRGILEDEQMYQ

PB1 VRKMMTNSQDTEISF

PB1 TNSQDTEISFTITGD

PB1 TEISFTITGDNTKWN

PB1 ENQNPRMFLAMITYI

PB1 KKKIEKIRPLLIDGT

PB1 KIRPLLIDGTASLSP

PB1 LIDGTASLSPGMMMG

PB1 SMELPSFGVSGVNES

PB1 SFGVSGVNESADMSI

PB1 GVNESADMSIGVTVI

PB1 KNNMINNDLGPATAQ

PB1 NNDLGPATAQMALQL

PB1 PATAQMALQLFIKDY

PB1 MALQLFIKDYRYTYR

PB1 FIKDYRYTYRCHRGD

PB1 RYTYRCHRGDTQIQT

PB1 CHRGDTQIQTRRSFE

PB1 VSDGGPNLYNIRNLH

PB1 PNLYNIRNLHIPEVC

PB1 NRSILNTSQRGILED

PB1 NTSQRGILEDEQMYQ

PB2 EKVERLKHGTFGPVHFR

PB2 MGMIGILPDMTPSTEMS

PB2 VVVSIDRFLRVRDQRGN

PB2 RFLRVRDQRGNVLLSPE

PB2 KTYFEKVERLKHGTF

PB2 KVERLKHGTFGPVHF

PB2 VSIDRFLRVRDQRGN

PB2 FLRVRDQRGNVLLSP

NS1 APFLDRLRRDQKSLKGR

NS1 PFLDRLRRDQKSLKG

NS2 SSFQDILMRMSKMQLGS

NS2 DILMRMSKMQLGSSS

NS2 ENSFEQITFMQALQL

M1 VFAGKNTDLEALMEWLK

M1 TDLEALMEWLKTRPILS

M1 FVQNALNGNGDPNNMDR

M1 KNTDLEALMEWLKTR

M1 EALMEWLKTRPILSP

M1 NGNGDPNNMDRAVKL

M2 KRGPSTEGVPESMREEY

M2 KRGPSTEGVPESMRE

NP IGRFYIQMCTELKLS

NP GKDPKKTGGPIYRRV

NP SNLNDATYQRTRALV

NP ATYQRTRALVRTGMD

NP NPAHKSQLVWMACHS

NP SQLVWMACHSAAFED

PA GDPNALLKHRFEIIE

PA EITGTMRKLADQSLP

PA LENFRAYVDGFEPNG

PA KFLLMDALKLSIEDP

PA DALKLSIEDPSHEGE

PA SIEDPSHEGEGIPLY

PA SHEGEGIPLYDAIKC

PA VLAELQDIENEEKIP

PA PEKVDFDDCKDVGDL

PA FDDCKDVGDLKQYDS

PA FNKACELTDSSWIEL

PA ELTDSSWIELDEIGE

PA SWIELDEIGEDVAPI

PA SVKEKDMTKEFFENK

PA DMTKEFFENKSETWP

PA FFENKSETWPIGESP

PB1 TTNTETGAPQLNPID

PB1 TIEVFRSNGLTANES

PB1 RSNGLTANESGRLID

PB1 TANESGRLIDFLKDV

PB1 GRLIDFLKDVMESMN

PB1 ITTHFQRKRRVRDNM

PB1 TQIPAEMLASIDLKY

PB1 DFALIVNAPNHEGIQ

PB1 VNAPNHEGIQAGVDR

PB1 HEGIQAGVDRFYRTC

PB1 EYDAVATTHSWIPKR

PB1 ATTHSWIPKRNRSIL

PB1 WIPKRNRSILNTSQR

PB1 ICSTIEELRRQK

PB2 IKKYTSGRQEKNPAL

PB2 SGRQEKNPALRMKWM

PB2 SDRVMVSPLAVTWWN

PB2 EEEVLTGNLQTLKIR

PB2 TGNLQTLKIRVHEGY

PB2 TLKIRVHEGYEEFTM

PB2 VHEGYEEFTMVGRRA

PB2 EEFTMVGRRATAILR

PB2 VGRRATAILRKATRR

PB2 LIQLIVSGRDEQSIA

PB2 VSGRDEQSIAEAIIV

PB2 EQSIAEAIIVAMVFS

PB2 EAIIVAMVFSQEDCM

PB2 AMVFSQEDCMIKAVR

PB2 QEDCMIKAVRGDLNF

PB2 IKAVRGDLNFVNRAN

PB2 RHFQKDAKVLFQNWG

PB2 SKMGVDEYSSTERVV

PB2 DEYSSTERVVVSIDR

PB2 MWEINGPESVLVNTY

PB2 GPESVLVNTYQWIIR

PB2 GNSPVFNYNKATKRL

PB2 FNYNKATKRLTVLGK

NS1 LETLILLRAFTEEGA

NS2 RNEKWREQLGQKFEE

M1 TEVETYVLSIIPSGP

M1 EVETYVLSIIPSGPL

M1 YVLSIIPSGPLKAEI

M1 VLSIIPSGPLKAEIA

M1 IPSGPLKAEIAQRLE

M1 GLVCATCEQIADSQH

M1 LVCATCEQIADSQHR

M1 TCEQIADSQHRSHRQ

M1 CEQIADSQHRSHRQM

M1 AMRTIGTHPSSSAGL

M1 MRTIGTHPSSSAGLK

M2 RLFFKCIYRRFKYGL

M2 LFFKCIYRRFKYGLK

HA CIGYHANNSTDTVDT

HA ANNSTDTVDTVLEKN

HA DTVDTVLEKNVTVTH

HA VLEKNVTVTHSVNLL

HA EELREQLSSVSSFER

HA QLSSVSSFERFEIFP

HA RNIPSIQSRGLFGAI

HA NIPSIQSRGLFGAIA

HA IQSRGLFGAIAGFIE

HA QSRGLFGAIAGFIEG

HA LFGAIAGFIEGGWTG

HA FGAIAGFIEGGWTGM

HA AGFIEGGWTGMVDGW

HA GFIEGGWTGMVDGWY

HA GGWTGMVDGWYGYHH

HA GWTGMVDGWYGYHHQ

HA MVDGWYGYHHQNEQG

HA VDGWYGYHHQNEQGS

HA YGYHHQNEQGSGYAA

HA GYHHQNEQGSGYAAD

HA ITNKVNSVIEKMNTQ

HA VNSVIEKMNTQFTAV

HA NSVIEKMNTQFTAVG

HA EKMNTQFTAVGKEFN

HA WTYNAELLVLLENER

HA TYNAELLVLLENERT

HA KNNAKEIGNGCFEFY

HA NNAKEIGNGCFEFYH

HA CMESVKNGTYDYPKY

HA SLGAISFWMCSNGSL

HA LGAISFWMCSNGSLQ

HA SFWMCSNGSLQCRIC

HA FWMCSNGSLQCRICI

HA SNGSLQCRICI

HA NGSLQCRICI

HA HANNSTDTVDTVLEKNV

HA DTVDTVLEKNVTVTHSV

HA DYEELREQLSSVSSFER

HA EQLSSVSSFERFEIFPK

HA LRNIPSIQSRGLFGAIA

HA IQSRGLFGAIAGFIEGG

HA FGAIAGFIEGGWTGMVD

HA FIEGGWTGMVDGWYGYH

HA TGMVDGWYGYHHQNEQG

HA WYGYHHQNEQGSGYAAD

HA TNKVNSVIEKMNTQFTA

HA VIEKMNTQFTAVGKEFN

HA IWTYNAELLVLLENERT

HA LKNNAKEIGNGCFEFYH

HA MESVKNGTYDYPKYSEE

HA SLGAISFWMCSNGSLQ

HA SFWMCSNGSLQCRICI

NA KGDVFVIREPFISCS

NA TFFLTQGALLNDKHS

NA LTIGISGPDNGAVAV

NA SGPDNGAVAVLKYNG

NA VCRDNWHGSNRPWVS

NA WHGSNRPWVSFNQNL

NA GYSGSFVQHPELTGL

NA FVQHPELTGLDCIRP

NA ELTGLDCIRPCFWVE

NA TIWTSGSSISFCGVN

NA IWTSGSSISFCGVNS

NA WSWPDGAELPFT

NA IGSKGDVFVIREPFIS

NA RTFFLTQGALLNDKHSN

NA ISGPDNGAVAVLKYNGI

NA VCRDNWHGSNRPWVSFN

NA WSGYSGSFVQHPELTGL

NA SFVQHPELTGLDCIRP

NA PELTGLDCIRPCFWVEL

NP MASQGTKRSYEQMET

NP MVLSAFDERRNKYLE

NP FDERRNKYLEEHPSA

NP NKYLEEHPSAGKDPK

NP EHPSAGKDPKKTGGP

NP TRALVRTGMDPRMCS

NP RTGMDPRMCSLMQGS

NP PRMCSLMQGSTLPRR

NP LMQGSTLPRRSGAAG

NP TLPRRSGAAGAAVKG

NP MIKRGINDRNFWRGE

NP RMCNILKGKFQTAAQ

NP RSALILRGSVAHKSC

NP LRGSVAHKSCLPACV

NP GKLSTRGVQIASNEN

NP LRSRYWAIRTRSGGN

NP VPSFDMSNEGSYFFG

NP MSNEGSYFFGDNAEE

NP VLSAFDERRNKYLEEHP

NP ERRNKYLEEHPSAGKDP

NP LEEHPSAGKDPKKTGGP

NP TRALVRTGMDPRMCSLM

NP TGMDPRMCSLMQGSTLP

NP MCSLMQGSTLPRRSGAA

NP GSTLPRRSGAAGAAVKG

NP FLARSALILRGSVAHKS

NP LILRGSVAHKSCLPACV

NP PRGKLSTRGVQIASNEN

NP TLELRSRYWAIRTRSGG

NP PIVPSFDMSNEGSYFFG

NP DMSNEGSYFFGDNAEEY

PA MEDFVRQCFNPMIVE

PA ETNKFAAICTHLEVC

PA AAICTHLEVCFMYSD

PA EKPKFLPDLYDYKEN

PA LPDLYDYKENRFIEI

PA DYKENRFIEIGVTRR

PA RFIEIGVTRREVHIY

PA GVTRREVHIYYLEKA

PA EVHIYYLEKANKIKS

PA YLEKANKIKSEKTHI

PA NKIKSEKTHIHIFSF

PA EKTHIHIFSFTGEEM

PA HIFSFTGEEMATKAD

PA TGEEMATKADYTLDE

PA ATKADYTLDEESRAR

PA YTLDEESRARIKTRL

PA ESRARIKTRLFTIRQ

PA IKTRLFTIRQEMASR

PA LKWALGENMAPEKVD

PA GENMAPEKVDFDDCK

PA DEIGEDVAPIEHIAS

PA DVAPIEHIASMRRNY

PA SHCRATEYIMKGVYI

PA TEYIMKGVYINTALL

PA KGVYINTALLNASCA

PA NTALLNASCAAMDDF

PA NASCAAMDDFQLIPM

PA AMDDFQLIPMISKCR

PA QLIPMISKCRTKEGR

PA ISKCRTKEGRRKTNL

PA TKEGRRKTNLYGFII

PA RKTNLYGFIIKGRSH

PA YGFIIKGRSHLRNDT

PA KGRSHLRNDTDVVNF

PA LRNDTDVVNFVSMEF

PA DVVNFVSMEFSLTDP

PA VSMEFSLTDPRLEPH

PA SLTDPRLEPHKWEKY

PA RLEPHKWEKYCVLEI

PA QVSRPMFLYVRTNGT

PA MFLYVRTNGTSKIKM

PA RTNGTSKIKMKWGME

PA SKIKMKWGMEMRRCL

PA KWGMEMRRCLLQSLQ

PA MRRCLLQSLQQIESM

PA LQSLQQIESMIEAES

PA QIESMIEAESSVKEK

PA IEAESSVKEKDMTKE

PA VCRTLLAKSVFNSLY

PA LAKSVFNSLYASPQL

PA FNSLYASPQLEGFSA

PA ASPQLEGFSAESRKL

PA EGFSAESRKLLLIVQ

PA ESRKLLLIVQALRDN

PA LLIVQALRDNLEPGT

PA ALRDNLEPGTFDLGG

PA LEPGTFDLGGLYEAI

PA FDLGGLYEAIEECLI

PA LYEAIEECLINDPWV

PA EECLINDPWVLLNAS

PA NDPWVLLNASWFNSF

PA LLNASWFNSFLTHAL

PA MEDFVRQCFNPMIVELA

PA IETNKFAAICTHLEVCF

PA AAICTHLEVCFMYSDFH

PA KPKFLPDLYDYKENRFI

PA DLYDYKENRFIEIGVTR

PA ENRFIEIGVTRREVHIY

PA IGVTRREVHIYYLEKAN

PA EVHIYYLEKANKIKSEK

PA LEKANKIKSEKTHIHIF

PA IKSEKTHIHIFSFTGEE

PA HIHIFSFTGEEMATKAD

PA FTGEEMATKADYTLDEE

PA ATKADYTLDEESRARIK

PA TLDEESRARIKTRLFTI

PA RARIKTRLFTIRQEMAS

PA TSQLKWALGENMAPEKV

PA ALGENMAPEKVDFDDCK

PA WIELDEIGEDVAPIEHI

PA IGEDVAPIEHIASMRRN

PA HCRATEYIMKGVYINTA

PA YIMKGVYINTALLNAS

PA VYINTALLNASCAAMDD

PA LLNASCAAMDDFQLIPM

PA AAMDDFQLIPMISKCRT

PA FQLIPMISKCRTKEGRR

PA ISKCRTKEGRRKTNLYG

PA KEGRRKTNLYGFIIKGR

PA TNLYGFIIKGRSHLRND

PA IIKGRSHLRNDTDVVNF

PA HLRNDTDVVNFVSMEFS

PA DVVNFVSMEFSLTDPRL

PA SMEFSLTDPRLEPHKWE

PA TDPRLEPHKWEKYCVLE

PA SRPMFLYVRTNGTSKIK

PA YVRTNGTSKIKMKWGME

PA TSKIKMKWGMEMRRCLL

PA KWGMEMRRCLLQSLQQI

PA RRCLLQSLQQIESMIEA

PA SLQQIESMIEAESSVKE

PA SMIEAESSVKEKDMTKE

PA VCRTLLAKSVFNSLYAS

PA AKSVFNSLYASPQLEGF

PA SLYASPQLEGFSAESRK

PA PQLEGFSAESRKLLLIV

PA SAESRKLLLIVQALRDN

PA LLLIVQALRDNLEPGTF

PA ALRDNLEPGTFDLGGLY

PA EPGTFDLGGLYEAIEE

PA DLGGLYEAIEECLINDP

PA EAIEECLINDPWVLLNA

PA LINDPWVLLNASWFNSF

PB1 NAISTTFPYTGDPPY

PB1 TFPYTGDPPYSHGTG

PB1 GDPPYSHGTGTGYTM

PB1 SHGTGTGYTMDTVNR

PB1 TGYTMDTVNRTHQYS

PB1 TGAPQLNPIDGPLPE

PB1 LNPIDGPLPEDNEPS

PB1 GPLPEDNEPSGYAQT

PB1 DNEPSGYAQTDCVLE

PB1 GYAQTDCVLEAMAFL

PB1 DCVLEAMAFLEESHP

PB1 AMAFLEESHPGIFEN

PB1 MEVVQQTRVDKLTQG

PB1 QTRVDKLTQGRQTYD

PB1 KLTQGRQTYDWTLNR

PB1 RQTYDWTLNRNQPAA

PB1 WTLNRNQPAATALAN

PB1 NQPAATALANTIEVF

PB1 TALANTIEVFRSNGL

PB1 ALTLNTMTKDAERGK

PB1 TMTKDAERGKLKRRA

PB1 AERGKLKRRAIATPG

PB1 LKRRAIATPGMQIRG

PB1 IATPGMQIRGFVYFV

PB1 MQIRGFVYFVETLAR

PB1 FVYFVETLARSICEK

PB1 ETLARSICEKLEQSG

PB1 SICEKLEQSGLPVGG

PB1 LEQSGLPVGGNEKKA

PB1 LPVGGNEKKAKLANV

PB1 NEKKAKLANVVRKMM

PB1 KLANVVRKMMTNSQD

PB1 TITGDNTKWNENQNP

PB1 NTKWNENQNPRMFLA

PB1 PIMFSNKMARLGKGY

PB1 NKMARLGKGYMFESK

PB1 ASLSPGMMMGMFNML

PB1 GMMMGMFNMLSTVLG

PB1 MFNMLSTVLGVSILN

PB1 YWWDGLQSSDDFALI

PB1 LQSSDDFALIVNAPN

PB1 EFTSFFYRYGFVANF

PB1 FYRYGFVANFSMELP

PB1 FVANFSMELPSFGVS

PB1 ADMSIGVTVIKNNMI

PB1 GVTVIKNNMINNDLG

PB1 IRNLHIPEVCLKWEL

PB1 FEKFFPSSSYRRPVG

PB1 PSSSYRRPVGISSMV

PB1 RRPVGISSMVEAMVS

PB1 ISSMVEAMVSRARID

PB1 PAQNAISTTFPYTGDPP

PB1 STTFPYTGDPPYSHGTG

PB1 TGDPPYSHGTGTGYTMD

PB1 SHGTGTGYTMDTVNRTH

PB1 TGAPQLNPIDGPLPEDN

PB1 NPIDGPLPEDNEPSGYA

PB1 LPEDNEPSGYAQTDCVL

PB1 PSGYAQTDCVLEAMAFL

PB1 TDCVLEAMAFLEESHPG

PB1 AMAFLEESHPGIFENS

PB1 VQQTRVDKLTQGRQTYD

PB1 DKLTQGRQTYDWTLNRN

PB1 RQTYDWTLNRNQPAATA

PB1 TLNRNQPAATALANTIE

PB1 PAATALANTIEVFRSNG

PB1 ALTLNTMTKDAERGKLK

PB1 MTKDAERGKLKRRAIAT

PB1 RGKLKRRAIATPGMQIR

PB1 RAIATPGMQIRGFVYFV

PB1 GMQIRGFVYFVETLARS

PB1 FVYFVETLARSICEKLE

PB1 TLARSICEKLEQSGLPV

PB1 CEKLEQSGLPVGGNEKK

PB1 SGLPVGGNEKKAKLANV

PB1 GNEKKAKLANVVRKMMT

PB1 KLANVVRKMMTNSQDTE

PB1 SFTITGDNTKWNENQNP

PB1 DNTKWNENQNPRMFLAM

PB1 APIMFSNKMARLGKGYM

PB1 TASLSPGMMMGMFNMLS

PB1 GMMMGMFNMLSTVLGVS

PB1 FNMLSTVLGVSILNLGQ

PB1 YWWDGLQSSDDFALIVN

PB1 TFEFTSFFYRYGFVANF

PB1 FFYRYGFVANFSMELPS

PB1 ESADMSIGVTVIKNNMI

PB1 CCNLFEKFFPSSSYRRP

PB1 KFFPSSSYRRPVGISSM

PB1 SYRRPVGISSMVEAMVS

PB1 GISSMVEAMVSRARIDA

PB2 MSQSRTREILTKTTV

PB2 TREILTKTTVDHMAI

PB2 TKTTVDHMAIIKKYT

PB2 DHMAIIKKYTSGRQE

PB2 MIPERNEQGQTLWSK

PB2 KHGTFGPVHFRNQVK

PB2 GPVHFRNQVKIRRRV

PB2 HADLSAKEAQDVIME

PB2 AKEAQDVIMEVVFPN

PB2 DVIMEVVFPNEVGAR

PB2 VVFPNEVGARILTSE

PB2 LMVAYMLERELVRKT

PB2 MLERELVRKTRFLPV

PB2 SVYIEVLHLTQGTCW

PB2 VLHLTQGTCWEQMYT

PB2 QGTCWEQMYTPGGEV

PB2 EQMYTPGGEVRNDDV

PB2 PGGEVRNDDVDQSLI

PB2 RNDDVDQSLIIAARN

PB2 DQSLIIAARNIVRRA

PB2 IAARNIVRRAAVSAD

PB2 IVRRAAVSADPLASL

PB2 AVSADPLASLLEMCH

PB2 PLASLLEMCHSTQIG

PB2 DILRQNPTEEQAVDI

PB2 GLRISSSFSFGGFTF

PB2 SSFSFGGFTFKRTSG

PB2 TAILRKATRRLIQLI

PB2 KATRRLIQLIVSGRD

PB2 GDLNFVNRANQRLNP

PB2 VNRANQRLNPMHQLL

PB2 QRLNPMHQLLRHFQK

PB2 MHQLLRHFQKDAKVL

PB2 NVMGMIGILPDMTPS

PB2 DQRGNVLLSPEEVSE

PB2 VLLSPEEVSETQGTE

PB2 EEVSETQGTEKLTIT

PB2 TQGTEKLTITYSSSM

PB2 KLTITYSSSMMWEIN

PB2 YSSSMMWEINGPESV

PB2 LYNKMEFEPFQSLVP

PB2 VRTLFQQMRDVLGTF

PB2 RILVRGNSPVFNYNK

PB2 VESAVLRGFLILGKE

PB2 PALSINELSNLAKGE

PB2 NELSNLAKGEKANVL

PB2 LAKGEKANVLIGQGD

PB2 KANVLIGQGDVVLVM

PB2 IGQGDVVLVMKRKRD

PB2 VVLVMKRKRDSSILT

PB2 KRKRDSSILTDSQTA

PB2 SSILTDSQTATKRIR

PB2 TDSQTATKRIRMAIN

PB2 DSQTATKRIRMAIN

PB2 SQSRTREILTKTTVDHM

PB2 EILTKTTVDHMAIIKKY

PB2 TVDHMAIIKKYTSGRQE

PB2 KHGTFGPVHFRNQVKIR

PB2 PGHADLSAKEAQDVIME

PB2 SAKEAQDVIMEVVFPNE

PB2 DVIMEVVFPNEVGARIL

PB2 VFPNEVGARILTSESQL

PB2 VAYMLERELVRKTRFLP

PB2 VYIEVLHLTQGTCWEQM

PB2 HLTQGTCWEQMYTPGGE

PB2 CWEQMYTPGGEVRNDDV

PB2 TPGGEVRNDDVDQSLII

PB2 RNDDVDQSLIIAARNIV

PB2 DQSLIIAARNIVRRAAV

PB2 AARNIVRRAAVSADPLA

PB2 RRAAVSADPLASLLEM

PB2 SADPLASLLEMCHSTQI

PB2 ILRQNPTEEQAVDICKA

PB2 SSFSFGGFTFKRTSGSS

PB2 RATAILRKATRRLIQLI

PB2 RKATRRLIQLIVSGRDE

PB2 GDLNFVNRANQRLNPMH

PB2 NRANQRLNPMHQLLRHF

PB2 LNPMHQLLRHFQKDAKV

PB2 DQRGNVLLSPEEVSETQ

PB2 LLSPEEVSETQGTEKLT

PB2 VSETQGTEKLTITYSSS

PB2 TEKLTITYSSSMMWEIN

PB2 TYSSSMMWEINGPESVL

PB2 LYNKMEFEPFQSLVPKA

PB2 FVRTLFQQMRDVLGTFD

PB2 LSINELSNLAKGEKANV

PB2 SNLAKGEKANVLIGQGD

PB2 EKANVLIGQGDVVLVMK

PB2 IGQGDVVLVMKRKRDSS

PB2 VLVMKRKRDSSILTDSQ

PB2 KRDSSILTDSQTATKRI

PB2 LTDSQTATKRIRMAIN

NS1 LLRAFTEEGAIVGEI

NS1 TEEGAIVGEISPLPS

NS1 IVGEISPLPSLPGHT

NS1 FTEEGAIVGEISPLPSL

NS2 REQLGQKFEEIRWLI

M1 WLKTRPILSPLTKGI

M1 LKTRPILSPLTKGIL

M1 PILSPLTKGILGFVF

M1 ILSPLTKGILGFVFT

M1 LTKGILGFVFTLTVP

M1 TKGILGFVFTLTVPS

M1 LGFVFTLTVPSERGL

M1 GFVFTLTVPSERGLQ

M1 TLTVPSERGLQRRRF

M1 LTVPSERGLQRRRFV

M1 SERGLQRRRFVQNAL

M1 ERGLQRRRFVQNALN

M1 QRRRFVQNALNGNGD

M1 RRRFVQNALNGNGDP

M1 VQNALNGNGDPNNMD

M1 NPLIRHENRMVLAST

M1 PLIRHENRMVLASTT

M1 HENRMVLASTTAKAM

M1 ENRMVLASTTAKAME

M1 VLASTTAKAMEQMAG

M1 LASTTAKAMEQMAGS

M1 TAKAMEQMAGSSEQA

M1 AKAMEQMAGSSEQAA

M1 EQMAGSSEQAAEAME

M1 QMAGSSEQAAEAMEV

M1 ENLQAYQKRMGVQMQ

M1 NLQAYQKRMGVQMQR

M1 YQKRMGVQMQRFK

M1 QKRMGVQMQRFK

M1 MEWLKTRPILSPLTKGI

M1 RPILSPLTKGILGFVFT

M1 LTKGILGFVFTLTVPSE

M1 GFVFTLTVPSERGLQRR

M1 TVPSERGLQRRRFVQNA

M1 GLQRRRFVQNALNGNGD

M1 TNPLIRHENRMVLASTT

M1 HENRMVLASTTAKAMEQ

M1 LASTTAKAMEQMAGSSE

M1 KAMEQMAGSSEQAAEAM

M1 LENLQAYQKRMGVQMQR

M1 YQKRMGVQMQRFK
